# Interventions supporting the empowerment of parent carers of children with neurodisability and other long‐term health conditions: A scoping review

**DOI:** 10.1111/dmcn.70039

**Published:** 2025-10-26

**Authors:** Jim Reeder, Morwenna Rogers, Phillip Harniess, Fatema Shamsaddin, Caomhán McGlinchey, Jane R Smith, Sally Kendall, Christopher Morris

**Affiliations:** ^1^ PenCRU (Peninsula Childhood Disability Research Unit), Health & Community Sciences University of Exeter Medical School Exeter UK; ^2^ Evidence Synthesis Team, NIHR ARC South West Peninsula University of Exeter Medical School Exeter UK; ^3^ Public Health & Sports Sciences, University of Exeter Medical School University of Exeter Exeter UK; ^4^ Exeter Collaboration for Academic Primary Care (APEx), Health & Community Sciences, University of Exeter Medical School University of Exeter Exeter UK; ^5^ Centre for Health Services Studies University of Kent Canterbury UK

## Abstract

**Aim:**

To compile information about interventions that have been developed to support the empowerment of parent carers of children and young people aged 0 to 19 years with neurodisability (e.g. cerebral palsy, epilepsy, autism) or other long‐term health conditions (e.g. asthma, diabetes, cancer).

**Method:**

Seven electronic databases and grey literature were systematically searched for potentially eligible studies and information sources. Identified sources were screened by two independent reviewers. Data were extracted using a custom tool developed by the review team, before being coded and recorded in an interactive online database. Eligibility criteria were inclusive to capture a broad range of interventions designed to address any component of parent carer empowerment.

**Results:**

A total of 212 information sources documenting 145 interventions were included in the review and are presented in the database (https://eppi.ioe.ac.uk/eppi‐vis/Review/Index/762). Parent carer‐focused interventions have been developed targeting a range of aspects of empowerment; however, there were issues with implementation, sustainability, and scalability.

**Interpretation:**

Many interventions have been designed to improve parent carer empowerment, targeting different aspects of parent carer behaviour. Designing further parent carer‐focused interventions may not be an efficient use of limited resources. We recommend that future research should prioritize adaptation, implementation, and robust evaluation of existing interventions, or address other modifiable influences on parent carer empowerment.

## Graphical abstract

This scoping review identified 145 different interventions designed to support parent carer empowerment. These interventions have been catalogued and are presented in an interactive, online database.

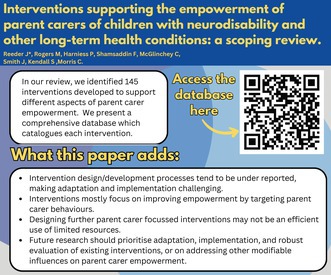

**Abbreviation**
TIDiER: Template for Intervention Description and Replication

**What this paper adds**

- Develops a comprehensive database cataloguing 145 interventions supporting parent carer empowerment.
- Intervention design/development processes tend to be underreported, making adaptation and implementation challenging.
- Interventions mostly focus on improving empowerment by targeting parent carer behaviours.

For more than 35 years health policymakers have advocated for patients and service users to take an active and participatory role in their own health care.^1^ The World Health Organization describes the process of patients strengthening their influence and control over the decisions and actions affecting their care as one of empowerment.^2^ Empowerment is especially important for people with long‐term health conditions and their carers, because they often need to interact with and navigate complex health care systems.

A long‐term health condition is defined as ‘a health problem that requires continuous management over a period of years or decades, and is one that cannot currently be cured but can be controlled with the use of medication and/or other therapies’.^3^ Examples of childhood‐onset long‐term health conditions can include chronic conditions such as asthma, diabetes, and cancer, as well as conditions typically categorized as neurodisability, such as cerebral palsy, epilepsy, and autism. In the UK there are nearly 1.7 million children and young people with a long‐term health condition,^4^ and it is thought that children and young people with neurodisability make up the highest proportion of this group.^5^

Parents of children and young people with neurodisability and other long‐term health conditions are rightly regarded as service users, as they actively seek to access health care and advocate for their child. A parent carer is defined in UK legislation as ‘any person aged 18 or over who provides or intends to provide care for a disabled child for whom the person has parental responsibility’.^6^

Parent carer empowerment can be described as the strengthening of parental influence and control over decisions and actions affecting their child's care. It is believed that promoting parent carer empowerment can improve parental quality of life,^7^ reduce reliance on health professionals,^8^ and improve the cost‐effectiveness of health services.^9^ In addition, although limited, there is also evidence to suggest that empowerment strategies might also improve health outcomes for children.^10^ This suggestion is underpinned by the World Health Organization's International Classification of Functioning, Disability and Health (ICF),^11^ which formally recognizes the impact of environmental and personal factors (including family dynamics) on the child's health.

Values and principles underpinning parent carer empowerment are intrinsic to contemporary child health and social care policy, where there is an explicit commitment to supporting children and their families to develop the requisite capacities to take control of decisions and actions affecting their own lives.^6^, ^12^, ^13^ Principles of parent carer empowerment are also a fundamental part of family‐centred care, which has long been the goal of child health care services both nationally and internationally.^14^

Significant effort has been made to try to better understand parent carer empowerment,^15^ and patient empowerment more generally.^9^, ^16^, ^17^ Despite this, conceptual frameworks and definitions for parent carer empowerment are often complex and ambiguous. Various phenomena/constructs have been proposed as potential components of empowerment. These have been interpreted as either processes (how people become empowered), contextual influences (which affect their readiness to be empowered), or outcomes (the consequences of empowerment).^9^, ^15^, ^16^, ^18^ Examples of components of empowerment include concepts such as self‐efficacy, self‐determination, self‐management, advocacy, health literacy, well‐being, shared decision‐making, and active coping.

Several parent carer‐focused interventions have been developed, which aim to support different components of empowerment. Indeed, evidence synthesis studies have reviewed the effectiveness of interventions targeting parent carer self‐efficacy,^19^ well‐being,^20^ mental health,^21^ and participation in shared decision‐making.^22^ However, there do not seem to be any studies that draw together all of this evidence to describe and catalogue the broad range of interventions designed to support all aspects of parent carer empowerment. To address this gap a scoping review was chosen as the most appropriate strategy, as recommended by published methodological guidance.^23^ This decision also aligns with the most up‐to‐date guidance from the JBI, which suggests that a scoping review is best suited to synthesize a large body of complex and/or heterogenous evidence.^24^ This approach helped to ensure that an appropriate breadth of evidence and information was identified; including evidence from quantitative, qualitative, and mixed‐methods studies, and evidence that falls outside traditional academic searches.

The aim of this scoping review was to determine the extent of the available evidence related to interventions that support parent carer empowerment, and to describe and catalogue any interventions identified. This included reporting how and where interventions have been designed, implemented, and evaluated. The goal was to provide a comprehensive overview of this topic, identifying knowledge gaps and offering recommendations for future work; as well as creating a practical resource for academics and health service providers who are developing new services and/or interventions for parent carers.

A preliminary search of MEDLINE (Ovid), PROSPERO (International Prospective Register of Systematic Reviews), and the Cochrane Database of Systematic Reviews (Cochrane Library) was conducted and no current or ongoing systematic reviews or scoping reviews on the topic were identified.

## METHOD

This scoping review used the methodological framework described by Arksey and O'Malley and refined by Levac et al.,^25^, ^26^ and is reported according to the Preferred Reporting Items for Systematic Reviews and Meta‐Analyses extension for Scoping Reviews (PRISMA‐ScR) Checklist.^27^ The protocol was registered and published on the Open Science Framework: https://doi.org/10.17605/OSF.IO/XEDBV.

### Involvement of people with lived experience

This scoping review was conducted as part of a continuing project related to understanding and supporting parent carer empowerment. The project is being delivered in partnership with a group of parent carer research partners. The group were involved in establishing the overarching aims of the review, discussing findings, and testing the usability and potential utility of the database. The group's involvement has led to exploration of alternative avenues, additional insights, and deepened understandings of the findings. This is described in greater detail in a completed GRIPP2‐SF (Table S1).

### Eligibility criteria

Studies were selected with eligibility criteria developed by the research team using the participant, concept, context model, as suggested in the JBI guidance.^24^ The eligibility criteria were purposefully broad and inclusive to provide a clear indication of the volume of evidence related to interventions supporting parent carer empowerment.

#### Participants

Sources of information about interventions for parent carers of children and young people aged up to 19 years of age with any long‐term health condition were eligible for inclusion. While our initial and prevailing focus was on parent carers of children with neurodisability, we felt a broader focus might help to identify interventions developed for parent carers of children with other long‐term conditions (e.g. asthma, diabetes, cancer) that may be used by parent carers of children with neurodisability. Being purposively inclusive ensured we could offer a more complete account of the state of the evidence related to interventions for parent carer empowerment.

Studies and sources of information that included parent carers of children and young people with wider age ranges were eligible for inclusion, if data for parent carers of children and young people aged 0 to 19 years were reported separately (e.g. in subgroup analysis) and could be extracted.

#### Concept

All sources of information about the design, delivery, and/or evaluation of interventions with the aim of supporting the empowerment of parent carers were eligible for inclusion. Given the broad nature of the concept of empowerment, this included information about any intervention that had the aim of supporting parent carers to improve their own health, participation, and/or function. An intervention is defined as ‘an act performed for, with or on behalf of a person or population whose purpose is to assess, improve, maintain, promote, or modify health, functioning or health conditions’.^28^

#### Context

Sources of information about interventions delivered both in‐person and/or online, in health care (primary care, community, or outpatient) and non‐health care settings, were eligible for inclusion. Sources of information about interventions delivered in an inpatient setting to support children/families negotiating an acute episode of care were excluded.

#### Information sources

This review included any published sources of information about the design, delivery, and/or evaluation of parent carer‐focused interventions. All study designs were considered. Systematic reviews were excluded; however, reference lists were hand searched and potentially relevant studies were considered using the eligibility criteria. There were no exclusion criteria related to date of publication or language of publication.

### Search strategy

#### Primary search strategy

Supported by an information specialist (MR), an initial limited search of MEDLINE (Ovid) was undertaken to identify articles on the topics of parent carer and patient empowerment. This helped the team to bring together a set of relevant terms to inform the development of a comprehensive search strategy (Appendix S1). The search strategy consisted of four groups of keywords and index terms: group 1, participant component (parent carers); group 2, intervention component (empowerment); group 3, context component A (children); group 4, context component B (long‐term childhood health conditions).

In group 4, as well as generic terms, we included examples of long‐term childhood health conditions that are either routinely used exemplars (e.g. cerebral palsy for movement disorders) or conditions with highest prevalence (e.g. asthma, epilepsy, neoplasms).

The search strategy, including all identified keywords and index terms, was adapted for each database and/or information source. The search was completed on 29th June 2023 and included the following electronic databases: MEDLINE (Ovid); CINAHL (EBSCOhost); Embase (Ovid); Applied Social Sciences Index and Abstracts (ProQuest); American Psychological Association (APA) PsycInfo (Ovid); ProQuest Dissertations and Theses Global (ProQuest).

#### Secondary search strategies

Reference lists of included studies and key papers were screened for additional studies. Forward citation checking of included papers was conducted. Experts (e.g. parent carer research partners, parent carer‐focused programme development teams, providers of parent carer‐led interventions), charities, and parent carer forums were consulted about existing interventions. This informed targeted searches through Google, to help ensure no relevant interventions were missed, particularly those that might not be reported in the academic literature. Only the first four pages of each Google search were screened

### Selection of sources of information

Citations from the electronic database searches were uploaded into Endnote (version 20.5; Clarivate Analytics, PA, USA) and duplicates were removed. Titles and abstracts were screened by two independent reviewers from the research team (JR, PH, FS, CMc) against the eligibility criteria. Any titles/abstracts not published in the primary language of the authors (English) were translated using a machine translation engine.

In a deviation from the study protocol, relevant studies/sources of information were retrieved in full and reassessed in detail against the eligibility criteria by just one reviewer (JR). Then all excluded studies were checked by a second independent reviewer (FS, PH, CMc, CM). This pragmatic decision was taken owing to the large number of information sources needing to be screened at full‐text stage (490) and the limited time and resources of the second reviewers. The strategy to double review only excluded studies is consistent with Cochrane guidance for rapid reviews, where time is a limiting factor in data management.^29^ Furthermore, the intention was for this to be an inclusive narrative review so there was less ‘risk’ involved in including additional studies.

Any disagreements that arose between the reviewers at each stage of the selection process were resolved through discussion and with a third independent reviewer if required (FS, PH, CMc, CM). Reasons for exclusion of sources of evidence at full‐text screening were recorded and are reported in Figure S1.

### Data management

#### Data extraction

Before data extraction, all included articles/sources of information related to each intervention were aggregated. Data were extracted for each intervention, with reference to all published sources where appropriate.

Data were extracted from included sources by one reviewer (JR) using a custom tool developed by the review team (Appendix S2). Ten per cent of extracted data were checked by a second reviewer (CM, SK, JRS). If key data were not provided in the available literature/evidence, further publications were sought and, when necessary, developers were contacted for additional details. Regular meetings were held between the review team to discuss any discrepancies in the data.

Extracted data included study or source characteristics (e.g. year, country, setting, source type) and details of how empowerment had been conceptualized, if available. Descriptive details about the intervention were recorded, using the Template for Intervention Description and Replication (TIDiER) framework as a guide (e.g. main aims, provider, mode of delivery, setting, structure, content, etc.).^30^ Any data related to the design, implementation, and evaluation of the intervention and any outcomes used to assess impact were also extracted.

As we did not seek to answer a research question about the effectiveness of identified interventions, meta‐synthesis of data and critical appraisal of methodological limitations or risk of bias were not performed. This is in line with current methodological guidance for conducting scoping reviews.^23^, ^24^, ^27^

#### Data synthesis

All included sources of information were uploaded to EPPI‐Reviewer 6 software.^31^ All extracted data associated with each intervention were coded using a bespoke tool, iteratively developed and tested by the review team during the data extraction process. Where possible, existing frameworks were used (e.g. TIDieR checklist^30^ and ICF browser^32^). This coding strategy was guided by the principles of intervention component analysis,^33^ which sets out a pragmatic approach for identifying the key features of complex interventions. By coding/categorizing the key components in this way, it allowed us to begin to compare different interventions that might otherwise seem disparate, and laid the foundation for more in‐depth analysis in the future. The taxonomy of the coding framework is available in Appendix S3.

## RESULTS

From the primary search, 7083 records were screened at the title and abstract stage. Including records identified through secondary search strategies, 490 full‐text sources of information were retrieved and screened. Of these, 212 sources of information documenting 145 discrete interventions were included in the review (Figure S1). A full list of references for the included sources of information sources is available in Appendix S4. This information, along with a comprehensive catalogue of all extracted data, is readily available in the online intervention database, which is an integral component of this paper.

### Intervention database

A substantive output of this scoping review is the online interactive database (https://eppi.ioe.ac.uk/eppi‐vis/login/open?webdbid=762), which comprehensively catalogues key intervention characteristics of all 145 interventions included in the review.

The database can be searched using the key intervention components as filters, either individually or combined. This supports the user in identifying interventions with shared characteristics, and in exploring their similarities and differences (e.g. only interventions offered to groups or only interventions evaluated using the Family Empowerment Scale).

A detailed explanation of how to use the database, including a worked example, is included in Appendix S5.

The database will remain live and will be updated annually by the lead author (JR). This will include adding new or missing interventions, and any missing/erroneous data about interventions that are already included should developers provide further information or evidence.

### Parent carer empowerment

Definitions and/or conceptualizations of parent carer empowerment were identified in the literature associated with 25% of the interventions (*n* = 35). There was no evidence of a shared, consensus definition, with authors citing a range of work on the topic, including the writings of Marc Zimmerman,^34^ Carl Dunst,^35^ Lia Fumagalli,^36^ Cheryl Gibson,^37^ Paul Koren,^38^ and Albert Friere.^39^ It is beyond the scope of this review to systematically synthesize this information. However, key ideas included parent carers gaining control and agency, parent carers participating in shared decision‐making, and parent carers developing the confidence and self‐esteem to meet the needs of their child and family. A central thread that ties these ideas together is the role and influence of power; specifically, how it is distributed, negotiated, and enacted in the therapeutic relationship.

### Characteristics of interventions

Characteristics of the interventions from all included sources of information are presented in Table 1.

**TABLE 1:** General characteristics of the interventions.

	Total number of interventions coded (*n* = 145)	Percentage of interventions (%)
**Main aim(s) of interventions**		
Empowerment	68	47
Health literacy	58	40
Well‐being	56	39
Self‐efficacy	55	38
Active coping	28	19
Self‐determination	27	19
Self‐management	14	10
Advocacy	13	9
Shared decision‐making	12	8
Autonomy	1	<1
Intrinsic motivation	0	0
**Intervention setting: geographical location**		
USA	53	37
UK	26	18
Australia	14	10
Canada	12	8
Iran	11	8
India	4	3
Ireland	4	3
The Netherlands	3	2
Japan	3	2
China	3	2
South Korea	2	1
Saudi Arabia	2	1
New Zealand	2	1
France	1	<1
Kenya	1	<1
Sweden	1	<1
Columbia	1	<1
Austria	1	<1
Iceland	1	<1
Turkey	1	<1
Belgium	1	<1
Taiwan	1	<1
Hong Kong	1	<1
Italy	1	<1
Ghana	1	<1
Pakistan	1	<1
Jordan	1	<1
**Setting type**		
Community	46	55
Clinical	80	32
Online	29	20
Not stated	2	1
**Child's primary condition/diagnosis**		
Neurodisability	93	64
Other – condition‐specific	34	24
Other – generic	18	12
**Neurodisability (*n* = 93)**		
Autistic spectrum disorder	48	52
Epilepsy	3	5
Cerebral palsy	5	3
Other – condition specific^a^	29	31
Other – generic	8	9
**Intervention provider**		
Academic research team	63	43
Service provider	65	45
Parent carer	33	23
Charity/not‐for‐profit	7	5
Academic research team and service provider	10	7
Academic research team and parent carer	5	3
Service provider and parent carer	9	6
**Intervention type**		
Parent carer‐focused	137	94
Parent carer‐mediated^b^	55	38
Social support	31	21
Staff training	2	1

^a^ Includes conditions such as traumatic brain injury (<1%; *n* = 1), fetal alcohol spectrum disorder (<1%; *n* = 1), brain tumours (1%; *n* = 2), and neurofibromatosis (<1%; *n* = 1).

^b^ Ninety‐five per cent of parent carer‐mediated interventions were hybrids with parent carer‐focused interventions.

Of the 145 interventions, almost half specified parent carer empowerment as one of their main aims (*n* = 68). Components of parent carer empowerment most commonly recorded as main aims of the interventions were improving parent carer health literacy (*n* = 58), well‐being (*n* = 56), and self‐efficacy (*n* = 55). Almost all of the interventions were either parent carer‐focused or had elements of their design that were intended to be parent carer‐focused (*n* = 137). Interventions that were not explicitly parent carer‐focused included staff training (*n* = 2), social support interventions (*n* = 4), and parent‐mediated interventions (i.e. interventions with a primary focus on teaching parents specific parenting skills and strategies they can implement with their children) (*n* = 3), all of which had clearly recorded parent carer‐focused outcomes.

Interventions have been developed and delivered in a broad range of geographical locations; however, nearly 75% (*n* = 105) were developed high‐income countries: USA, UK, Australia, and Canada. There was variability in the type of setting interventions delivered, with two‐thirds delivered outside the clinical setting, either in the community (*n* = 55) or online (*n* = 20). Most interventions were designed to be delivered by academic research teams and/or service providers (*n* = 128); 23% of interventions were delivered by parent carers either alone (*n* = 19) or in partnership with academics/service providers (*n* = 14).

Interventions have been developed for parent carers of children with a wide range of long‐term health conditions. Sixty‐four per cent were for parent carers of children with neurodisability (*n* = 93), with more than half of these specifically for parent carers of children with autism (*n* = 52). Other neurodisability diagnoses included epilepsy (3%; *n* = 5), cerebral palsy (2%; *n* = 3), and some more specific conditions such as traumatic brain injury (<1%; *n* = 1), fetal alcohol spectrum disorder (<1%; *n* = 1), brain tumours (1%; *n* = 2), and neurofibromatosis (<1%; *n* = 1). Of the remaining interventions, 18% were for parents of children with any long‐term health condition (*n* = 12) and 24% were for parents of children with a specified health condition not categorized as neurodisability (e.g. asthma, diabetes, kidney disease) (*n* = 34).

There has been an interest in interventions for parent carer empowerment from as early as 1985; however, most interventions identified have been developed in the past 11 years (74%; *n* = 107).

### Structure of interventions

Details about the structure of the interventions are presented in Table 2. Two‐thirds of interventions were delivered to a group (*n* = 93), with the remainder either delivered to individual parent carers (*n* = 35) or accessed independently by parent carers in their own time (*n* = 17). Most interventions were delivered face to face (*n* = 117); however, some of these also had an online/virtual offer (*n* = 26). Only 7% were solely delivered virtually (*n* = 11).

**TABLE 2:** Structure of the interventions.

	Total number of interventions coded (*n* = 145)	Percentage of interventions (%)
**Mode of delivery (how?)**		
Group	84	58
Individual	35	24
Independent	17	12
Group and individual	9	6
**Platform of delivery (how?)**		
Virtual	11	7
Face to face	91	63
Self‐paced online	17	12
Virtual and face to face	26	18
**Materials used (what?)**		
Shareable information (physical)	32	22
Shareable information (online)	62	43
Presentations	73	50
Intervention manual	1	<1
Digital tool	6	4
Other props	3	2
None specified	28	19
**Procedure (what?)**		
Information provision (synchronous)	119	82
Information provision (asynchronous)	114	79
Interactive activities (synchronous)	18	12
Interactive activities (asynchronous)	11	8
Advocacy work	14	10
**Content (what?)** ^a^		
Self‐care	43	30
Interpersonal interactions and relationships	42	29
Community, social, and civic life	16	11
Domestic life	106	73
Learning and applying knowledge	59	41
Specific content related to child's condition	28	19
None specified/needs‐based	17	12
**Evidence that the intervention is manualized?**		
Yes	43	30
No	102	70

^a^ Criteria are defined in the International Classification of Functioning, Disability and Health browser; see Appendix S4 for examples.

Most interventions consisted of information provision (*n* = 137) and interactive activities (*n* = 125). Information was shared using strategies including provision of written resources (*n* = 94) and presentations (*n* = 73). Of the 145 interventions, there was evidence that 30% (*n* = 43) were manualized, offering specific guidance for administration and delivery.

Content of the interventions was varied, and often covered multiple topics. Most common was content related to performing everyday tasks/activities, including care responsibilities for children (i.e. domestic life; *n* = 106). Only 19% of interventions included specific content related to the child's condition/diagnosis (*n* = 28).

### Design, implementation, and evaluation of interventions

Details about the design, implementation, and evaluation strategies, and any outcome measures used, are presented in Table 3. Data about the strategies used to design/develop interventions were limited in the sources of information identified, with only 10% of interventions having a published logic model (*n* = 14). Twenty‐six per cent of interventions included parent carers in the co‐design processes (*n* = 37). Data on implementation were also very limited and/or unclear, with only 13% of interventions presenting a clear strategy for implementation (*n* = 19).

**TABLE 3:** Design, implementation, and evaluation strategies.

	Total number of interventions coded (*n* = 145)	Percentage of interventions (%)
**Design strategy: is there clear evidence of coproduction with parent carers?**		
Yes	37	26
No	108	74
**Is there evidence of a published logic model?**		
Yes	14	10
No	131	90
**Implementation strategy: is there evidence of a clear implementation strategy?**		
Yes	19	13
No	126	87
**Evaluation strategy: type of study/approach used**		
Randomized controlled trial	31	21
Randomized controlled trial (protocol only)	3	2
Quasi‐experimental study	48	33
Pilot	31	21
Feasibility	16	11
Qualitative	18	12
Mixed methods	10	7
None identified	6	4
**Outcome measures** ^**a**^		
Family Empowerment Scale	31	22
Parenting Stress Index – Short Form	22	15
Parenting Sense of Competence Scale	10	7
Warwick Edinburgh Mental Health Wellbeing Scale	8	6
Depression Anxiety and Stress Scale	6	4
General Health Questionnaire	6	4
Paediatric Quality of Life Family Impact Module	6	4

^a^ Only the top seven most commonly used outcome measures are reported here. In total 133 different standardized outcome measures were used to evaluate the included interventions. A full list is included in the online database.

Interventions had been evaluated using a range of strategies, including randomized controlled trials (*n* = 31), quasi‐experimental studies (*n* = 48), and qualitative approaches (*n* = 18). One hundred and thirty‐three different standardized outcome measures were used in evaluation studies. The most common measure used was the Family Empowerment Scale (*n* = 31). Several studies also used non‐standardized measures developed to evaluate specific interventions (*n* = 78).

## DISCUSSION

This scoping review summarizes current evidence about the characteristics of interventions developed to support the empowerment of parent carers of children and young people with long‐term health conditions. We have presented a comprehensive interactive database, cataloguing 145 interventions, which we believe will be an important, practical resource for anyone searching for or planning to develop an intervention for parent carers. Below, we discuss the challenges that exist for stakeholders when trying to navigate the landscape of parent carer empowerment, and highlight areas for future work.

While our primary focus was on parent carers of children of neurodisability, we took the decision to broaden our search to include interventions that had been designed to empower parent carers of children with any long‐term disability. The rationale was to ensure we did not miss any information that might contribute to a more complete description of interventions for parent carer empowerment, and that might ultimately benefit parent carers of children with neurodisability.

This strategy led to the inclusion of 34 additional interventions (24% of all included interventions). Comparing the data from these interventions with the rest of the data set did not reveal any noteworthy differences; however, we believe the inclusion of these interventions has added to the richness and utility of the database. For example, one study in this subgroup presented a very clear example of the intervention development process, including publication of a logic model.^40^ In addition, other studies in this subgroup used outcome measures that had not been previously identified (e.g. The Family Management Measure, The Impact on the Family Scale, The Parental Health Questionnaire – Parent version, and The Perceived Efficacy in Parent Physician Interactions).

In this review we identified a very large number of interventions, developed in a variety of contexts, all of which have been designed to trigger a change that contributes to improved parent carer empowerment. With so many interventions, it can be difficult for policymakers, service providers, and parent carers themselves to decide what might work best, for whom, and in what context. We suggest several factors that might contribute to this difficulty, including (1) the complex nature and variability of the interventions; (2) the underreporting of the design and development processes, and procedural details of interventions; (3) inadequate implementation planning; and (4) the heterogeneity of outcome measures used for evaluation.

The UK's Medical Research Council suggests that an intervention is considered complex because of the properties of that intervention. This includes when an intervention has more components; when it is targeting a variety of behaviours in different groups or settings; when expertise and skills are required by those delivering or receiving it; and when there is a permitted level of flexibility in the intervention and/or its components.^41^ All interventions included in this review fit these criteria.

The interventions are complex, not only because of their internal properties but also because they are delivered in complex, changeable, and often unpredictable social systems.^42^ Critical realist thinking suggests that to understand whether the components (or mechanisms) of an intervention are likely to work, it is imperative to first understand the social context in which the intervention is embedded (including the different individuals, different providers, different settings, etc.).^43^ For interventions supporting the empowerment of parent carers of children with long‐term health conditions, the social context(s) in which they are delivered are likely to be highly complex and highly individualized. This begs the question, even if we have strong evidence that an intervention works for one individual or group in a specific context, how do we know whether it will work for everyone, or indeed anyone else? A potential answer is for intervention developers to be explicit in describing expected mechanisms of change and, importantly, how they are conditional and contingent on the local context in which they are delivered. This might be approached by publishing logic models or intervention theory.^42^ A good example is found in the Online Parent Information and Support (OPIS) literature.^40^ However, it is acknowledged that it can be incredibly difficult to identify all change mechanisms in such complex systems.^33^ This is especially true of childhood disability and illness contexts, where there are so many actors and agents at multiple levels in the system; and where change in one aspect of the system can alter social dynamics, giving rise to intended and unintended changes in other aspects of the system.

A large percentage of the interventions included in this study did not publish logic models or clearly articulated change mechanisms, and descriptions of context were limited. This lack of information leaves policymakers and service providers with significant uncertainty about the relevance, appropriateness, and applicability of the interventions to their setting/context. It also perhaps explains why there continues to be such proliferation of new interventions.

We suggest that there would be significant benefit from greater transparency in the reporting of the intervention design process. This might be facilitated by better use of existing reporting standards, such as the GUIdance for rEporting intervention Development (GUIDED) framework.^44^ Like other reporting standards, the GUIDED model offers a checklist for users to complete. This consists of 14 items, which include giving a clear explanation of the context for which the intervention is developed, and any changes/adaptions that might be required for different subgroups. More consistent use of such a tool would not only help to clearly articulate how and why an intervention should work but also improve potential for its adaptation and application in different contexts.

In addition to inconsistent reporting of the intervention design process, we also found underreporting of the procedural details of the interventions included in this review. In 2010 the CONsolidated Standards Of Reporting clinical Trials (CONSORT 2010) was published, which recommended that interventions should be described with sufficient detail to allow for replication.^45^ This was operationalized by the TIDieR checklist, which offers authors a standardized framework to report this detail.^30^ In our review, there were very few sources of information that published (or referenced) a completed TIDieR checklist. While it was often possible to extract much of the information in the checklist, there were still some gaps and a lack of clarity, leaving room for variable interpretations of procedure. A good example of a well‐described intervention using TIDieR is found in the ‘Healthy Mothers Healthy Families’ literature.^46^

More consistent reporting of the procedural detail of interventions would also support improved fidelity of intervention delivery. Acknowledging one of the key characteristics of a complex intervention, we suggest that, when reporting procedural detail, there would be great value in including information related to where flexibility might exist in an intervention, such that it might be adaptable to different contexts. The benefits and challenges of balancing fidelity and potential for adaptation/flexibility, and building this into intervention design processes, have been discussed in the literature.^47^

Despite the Medical Research Council's guidance, which encourages ‘deliberate efforts to increase impact and uptake of successfully tested health innovations’, there was very limited evidence of such efforts in the sources of information included in the review. Apart from a few exceptions (see Health Parent Carers implementation logic model),^48^ there was widespread omission of any information related to implementation, both in the design and evaluation strategies of the included interventions. This is a surprising finding given the increasing profile of implementation science as the key discipline underpinning the translation of research evidence into policy and clinical practice. A possible explanation is that there is a greater emphasis among intervention developers to pursue a traditional research pathway of scaled‐up effectiveness trials (e.g. randomized controlled trials) over other more pragmatic implementation work.

The Standards for Reporting Implementation studies (StaRI) have been published to support more consistent reporting of implementation studies.^49^ While the primary purpose of StaRI is to report on the implementation strategy, it also includes details about measuring the impact of the interventions. We suggest that this might be a useful framework to plan and undertake studies that combine both effectiveness and implementation processes. The potential benefits and application of such ‘hybrid’ study designs have been discussed in the literature.^50^

In the included sources of information, we identified huge variation in the outcome measures used to evaluate the effectiveness of the interventions. The most commonly used outcome was the Family Empowerment Scale;^38^ however, this was only used in 22% of the included interventions. This variation might be expected, given the multifaceted nature of parent carer empowerment and the broad variety of interventions included; however, there was still significant variation in outcomes used for interventions targeting more closely related behaviours. The lack of consistency in outcome measures used makes it difficult to synthesize effectiveness data about related studies, let alone about studies related to a more complex phenomena such as empowerment. We suggest that development of a core outcome set for parent carer empowerment would be a useful step forward to improve the quality and consistency of information about which interventions work or not. Furthermore, it may also offer greater consistency about how parent carer empowerment might be successfully operationalized.

When considering evaluating effectiveness of interventions, another strategy might be to use an intervention component analysis approach.^33^ This approach aims to identify how interventions (with similar aims) differ from one another and explores which of the differences seem to be important. We suggest that this approach is of particular use when programme theory is missing, or when it fails to account for variance in outcomes. In addition, there is a strong emphasis on knowledge translation, bridging the gap between evidence of effectiveness and practical implementation.

There is growing evidence that involving individuals with lived experience in research improves relevance, applicability, and success of that research.^51^, ^52^ Given the nature of parent carer empowerment, we suggest there is an even stronger moral and ethical imperative to ensure parent carers are fully involved in the design and development of interventions that support it. It is also recognized that there are likely to be often unmeasured participatory and emancipatory benefits to parent carers being involved in the design and/or delivery of such interventions, which should not be undervalued as important project outcomes (see ENVISAGE – reflections on a parent–researcher partnership).^53^

Only 26% of included interventions described involving parent carers in their design and development, and parent carers supported the delivery of 23% of interventions. Despite this, information related to the impact of involvement (i.e. any changes made to interventions as a consequence of involvement) was more limited. We suggest that this could be improved by more consistent use of reporting guidance (e.g. Guidance for Reporting Involvement of Patients and the Public – GRIPP2‐short form).^54^

It is important to acknowledge that parent carer empowerment is influenced by a complex system of contextual factors and that barriers to it can exist in different areas and with different actors (e.g. parent carers, health professionals, and service providers) of that system. Of the 145 included interventions, all but two^55^, ^56^ aimed to improve parent carer empowerment outcomes by targeting behaviour change of parent carers themselves. This potentially highlights a lack of acknowledgement and intention to address barriers to parent carer empowerment that exist in other areas and/or with other actors in the complex system. Indeed, a recent commentary has suggested that parent carers do not want or need empowerment interventions; rather, they want a health care system that does not disempower them.^57^ It is perhaps a little nihilistic to suggest services should stop offering interventions that target change in parent carer behaviour; this scoping review highlights several programmes that have had some very encouraging outcomes. In addition, it is likely that changes in parent carer behaviour will also have a ‘knock‐on’ effect, leading to changes in other areas of the complex system.^42^

It is recognized that there are some aspects of the complex system that are difficult to change, and others (such as parent carer behaviour) that are more modifiable and more likely to benefit from targeted intervention.^58^ It does makes sense, therefore, that limited resources have been committed to these ‘easy to change’ areas. Moving forwards, we suggest that more effort needs to be made to identify and address barriers to parent carer empowerment that exist in other areas and with other actors in the complex system. This may begin with a deeper exploration into how power is negotiated, distributed, and enacted in the complex system, to gain a more thorough understanding of how and where barriers to parent carer empowerment exist.^59^ Some work is already being done in this area by the ENVISAGE team, who are in the process of adapting their programme for service providers (ENVISAGE‐SP).

This scoping review has been reported according to PRISMA‐ScR standards. We have been comprehensive, robust, and transparent in our approach to searching and screening information sources and in extracting and managing data. We rigorously adhered to our published study protocol, clearly reporting any deviations from this plan.

We used recognized tools to code and categorize the extracted data (e.g. TIDieR checklist and ICF browser), incorporating contemporary ideas for identifying key components of the interventions. The key output of the intervention database is likely to be of significant interest to researchers and health professionals working in this field.

A limitation of this review is related to parent carer empowerment being such a poorly defined concept. Consequently, the review captured a heterogenous range of interventions related to empowerment. Furthermore, while significant effort was given to ensuring completeness, it may be there are interventions that have been missed, although these can be added to the online database if flagged. A further limitation is that no quality assessment of included information sources was completed. While it was not the intention of the scoping review to include this information, detail about research quality would have added value and further utility to the database. This is a potential avenue for future research in this area.

We make several recommendations and suggestions for future work. First, the database can be used as a resource to guide more focused, in‐depth investigation of interventions for parent carer empowerment; in particular, exploring and analysing which components of interventions effect the greatest and most relevant change for parent carers. Second, given the heterogeneity of interventions (both in design and evaluation) when exploring change mechanisms and effectiveness, it may be appropriate to consider contemporary strategies such as intervention component analysis. Third, intervention developers should provide greater clarity and consistency when reporting design and procedural details of the intervention. We suggest that this would support those wanting to adapt/implement exiting interventions. Fourth, new interventions could target change in other areas of the complex system; for example, changing the behaviour and actions of health professionals. Fifth, when involving people with lived experience in intervention development/evaluation/implementation work, we suggest that greater attention should be given to reporting the impact of that involvement by using existing reporting standards

## CONCLUSIONS

Very many interventions have been designed to improve parent carer empowerment, by targeting different aspects of parent carer behaviour. Given the plethora of existing interventions, designing further parent carer‐focused interventions may not be an efficient use of limited resources. It is recommended that future research should focus on the adaptation and/or implementation of existing interventions. However, owing to underreporting of design processes and procedural details (including expected change mechanisms), we acknowledge that it may be difficult for providers to know which of these interventions (or intervention components) will be most suitable (to adapt) for their specific context.

We also recommend that greater attention be given to developing interventions and/or policies that target change mechanisms in other areas, and with other actors, in the complex system of parent carer empowerment. To support this endeavour, further work is required to better understand this complex system, in particular the context‐specific influences, barriers, and enablers.

To do this work well, we suggest that parent carers should be fully involved at each stage of the process: from gaining a better understanding of the complex system and prioritizing change mechanisms, to designing/adapting, implementing, and delivering interventions. In addition to the well‐reported methodological and participatory benefits, there are also strong moral and ethical drivers for parent carers to have greater agency and influence in the research process, reflecting a broader commitment to parent carer empowerment.

## FUNDING INFORMATION

JR, clinical doctoral research fellow (NIHR302886), was funded by Health Education England/National Institute for Health and Care Research (NIHR) for this research project. The project was supported by the NIHR ARC Southwest Peninsula (known as PenARC). The views expressed in this publication are those of the author(s) and not necessarily those of the NIHR, NHS or the UK Department of Health and Social Care.

## Supporting information


**Table S1:** GRIPP2 SF – reporting on public involvement.

**Appendix S1:** Preliminary search strategy (for Medline [OVID]).

**Appendix S2:** Data extraction tool.

**Appendix S3:** Coding framework taxonomy.

**Appendix S4:** Reference list of included information sources.

**Appendix S5:** Using the database.

**Figure S1:** PRISMA flow chart.

**Data S1:** Preferred Reporting Items for Systematic reviews and Meta‐Analyses extension for Scoping Reviews (PRISMA‐ScR) Checklist

## Data Availability

The data that supports the findings of this study are available in the supplementary material of this article.
