# Supplementary material for: Interventions supporting the empowerment of parent carers of children with neurodisability and other long‐term health conditions: A scoping review
Source: Dev Med Child Neurol. 2025 Oct 26;68(4):489–500. doi: 10.1111/dmcn.70039 (PMC12982629; doi:10.1111/dmcn.70039)
Supplement: Supplementary file 1 — Table S1: GRIPP2 SF – reporting on public involvement. [file DMCN-68-489-s001.docx]

## Table S1: GRIPP2 SF – Reporting on Public Involvement

| Topic | Item |
| --- | --- |
| 1. Aim   (Report the aim of PPI in the study) | The group of parent carer research partners were encouraged to bring their own lived experience and expertise, with the aim to:   - Provide a clear understanding of the most meaningful components/factors of parent carer empowerment. - Ensure our scoping review captured and recorded information that was relevant to parent carers, as well as academics and service providers. - Explore alternative avenues, gain additional insights and offer potential different understandings of the findings. |
| 1. Methods   (Provide a clear description of the methods used for PPI in the study) | A group of five parent carer partners was convened to support all stages of the planning, delivery and dissemination of the overarching project. The group is from a diverse range of ethic backgrounds, living across the UK. There are four mothers and one father. This has been supported by the Family Involvement Co-ordinator (also a parent carer) linked to PenCRU’s Family Faculty at the University of Exeter.  The group met regularly via Zoom to discuss the progress of the review and to share their contributions. Tasks included facilitated discussion; designing, sharing and checking of materials (e.g. the eligibility criteria and the data extraction form); and piloting the database.  Provision was made to ensure the group had adequate access to necessary IT and training. The group were also compensated for their time and any costs incurred in line recommended policy/practice. |
| 1. Study Results   (Outcomes—Report the results of PPI in the study, including both positive and negative outcomes). | The PPI strengthened the study in a number of ways:   1. **Defining eligibility criteria.**  - Initially it was the intention to keep a focus on programmes designed to support empowerment of parent carers of children with neurodisability. The parent carer partners felt that it was important to broaden the criteria to include programmes designed for parent carers of children with other long-term disabilities (e.g. cancer, diabetes, etc…) as this would offer a more complete picture of empowerment programmes. - Parent carer partners were also very clear on what should not be included – specifically, programmes where there was only parent carer coaching and where there were no parent carer focussed outcomes.  1. **Data identification and extraction.**  - Early discussions about how empowerment has been conceptualised helped to inform the design of the data extraction form, and the subsequent coding of extracted data.  1. **Creation and utility of the database.**  - Parent carer partners trialled the database and gave suggestions for how to make it more accessible. - The group discussed the usefulness of the database for parent carers. It was agreed that parent carers are very unlikely to be primary users of the database. We did discuss if there should be any additional outputs from the study more tailored for parent carers; however, the group felt this was an unnecessary use of time/resource.  1. **Interpretations of findings.**  - Whilst it was not the intention of this study to offer an in depth discussion of the findings, the group did offer support to identify gaps and to make some recommendations for future work.   There were also some limitations to parent carer involvement:   1. **Screening sources of information.**  - Much of the included information has come from published scientific literature. This was often written in ways that was difficult to understand and the process was extremely time consuming. As such, parent carers were not involved in this process. |
| 1. Discussions and Conclusions   (Outcomes—Comment on the extent to which PPI influenced the study overall. Describe positive and negative effects) | We feel PPI in this study was effective and materially influenced important aspects of the study, based on the impacts in section 3.  This might have been related to several factors. Firstly the parent carer partners had received training, both associated with this project and with previous studies they had been involved with linked to their membership of the Family Faculty. In addition, the support of a dedicated Family Involvement Co-ordinator, helped to quickly establish a trusting relationship between the parent carer partners and the rest of the research team.  The correct processes were in place – beginning the partnership from the planning phase of the study and ensuring there was sufficient budget for appropriate compensation. There was also a commitment from the research team to a collaborative partnership with parent carers.  However, there were limitations. It is acknowledged that the systematic process of this review did limit possible input from parent carer partners on developing the methods for the study. Furthermore, the timescales involved in such an extensive search, did mean that parent carer partners were often not actively involved in the study (e.g. during the title/abstract and full text screening process). We did discuss parent carer partners involvement in these stages of the study; however the group felt that their ability to carry our screening was limited by difficulties reading and understanding academically written literature, and by the time commitment involved. We tried to mitigate for this by offering regular updates to the group during this time. |
| 1. Reflections/critical perspective   (Comment critically on the study, reflecting on the things that went well and those that did not, so others can learn from this experience) | We feel that PPI was embedded as far as is possible for a systematic scoping review.  We found it very helpful to use the Involvement Matrix as a tool to discuss and negotiate levels of involvement for this study, in the context of the larger project. Involving the parent carer partners throughout this early stage of the project helped to foster trusting relationships, which continue to bear fruit in ongoing work.  It is acknowledged that this level of involvement does require additional time and resources (in what is already a time intensive study); however, for the reasons identified in section 3 and 4, we believe this has been an appropriate use of resource.  A limitation is that all meetings have been held on Zoom and parent carer partners have never met in-person. Meeting face to face may have facilitated relationship building and potentially more diverse involvement. However, this form of involvement is very convenient for parent carers, and allows for involvement from more geographically diverse areas. |
