# Supplementary material for: Interventions supporting the empowerment of parent carers of children with neurodisability and other long‐term health conditions: A scoping review
Source: Dev Med Child Neurol. 2025 Oct 26;68(4):489–500. doi: 10.1111/dmcn.70039 (PMC12982629; doi:10.1111/dmcn.70039)
Supplement: Supplementary file 2 — Appendix S1: Preliminary search strategy (for Medline [OVID]). [file DMCN-68-489-s007.docx]

## Appendix S1: Preliminary Search Strategy (for Medline OVID)

| # | Searches | Results (29.06.23) |
| --- | --- | --- |
| 1 | parent.ab,ti. | 158144 |
| 2 | parents.ab,ti. | 207938 |
| 3 | parent carer.ab,ti. | 156 |
| 4 | caregiver.ab,ti. | 37602 |
| 5 | mother.ab,ti. | 126674 |
| 6 | mothers.ab,ti. | 165220 |
| 7 | father.ab,ti. | 27537 |
| 8 | fathers.ab,ti. | 25231 |
| 9 | guardian.ab,ti. | 3922 |
| 10 | "carer*".ab,ti. | 18475 |
| 11 | Parents/ | 80660 |
| 12 | 1 or 2 or 3 or 4 or 5 or 6 or 7 or 8 or 9 or 10 or 11 | 628897 |
| 13 | "empower*".ab,ti. | 37414 |
| 14 | health literacy.ab,ti. | 13165 |
| 15 | self-efficacy.ab,ti. | 39194 |
| 16 | self determination.ab,ti. | 6089 |
| 17 | shared decision making.ab,ti. | 12811 |
| 18 | active coping.ab,ti. | 1859 |
| 19 | (well being adj3 parent*).ab,ti. | 1328 |
| 20 | (well-being adj3 parent*).ab,ti. | 1328 |
| 21 | autonomy.ab,ti. | 37336 |
| 22 | self-management.ab,ti. | 25240 |
| 23 | intrinsic motivation.ab,ti. | 2215 |
| 24 | Empowerment/ | 750 |
| 25 | 13 or 14 or 15 or 16 or 17 or 18 or 19 or 20 or 21 or 22 or 23 or 24 | 162538 |
| 26 | 12 and 25 | 17267 |
| 27 | "child*".ab,ti. | 1622171 |
| 28 | Child/ or Infant/ or Adolescent/ | 3499510 |
| 29 | 27 or 28 | 3983316 |
| 30 | 26 and 29 | 12122 |
| 31 | "disab*".ab,ti. | 280770 |
| 32 | neurodisability.ab,ti | 313 |
| 33 | impairment.ab,ti. | 353946 |
| 34 | long term health condition.ab,ti. | 83 |
| 35 | complex needs.ab,ti. | 2142 |
| 36 | medical complexity.ab,ti. | 1054 |
| 37 | dysfunction.ab,ti. | 492411 |
| 38 | Intellectual Disability/ | 59736 |
| 39 | chronic disease/ | 281071 |
| 40 | Nervous System Diseases/ | 47656 |
| 41 | Autism Spectrum Disorder/ or Autistic Disorder/ | 41275 |
| 42 | cerebral palsy/ | 23621 |
| 43 | Epilepsy/ | 85125 |
| 44 | Asthma/ | 139276 |
| 45 | Diabetes Mellitus, Type 1/ | 85797 |
| 46 | Neoplasms/ | 499832 |
| 47 | 31 or 32 or 33 or 34 or 35 or 36 or 37 or 38 or 39 or 40 or 41 or 42 or 43 or 44 or 45 or 46 | 2225397 |
| 48 | 30 and 47 | 2373 |
