# Supplementary material for: Interventions supporting the empowerment of parent carers of children with neurodisability and other long‐term health conditions: A scoping review
Source: Dev Med Child Neurol. 2025 Oct 26;68(4):489–500. doi: 10.1111/dmcn.70039 (PMC12982629; doi:10.1111/dmcn.70039)
Supplement: Supplementary file 4 — Appendix S3: Coding framework taxonomy. [file DMCN-68-489-s005.docx]

## Appendix S3- Coding Framework Taxonomy

### Main aims

*Constructs used to code the ‘Main aim/s’ were derived from components of empowerment identified in the literature ^7, 10,11,13^ These were the same components used in group 2 of the primary search strategy. An additional over-arching code of ‘Empowerment’ was used when this was an explicit aim of the intervention. Interventions could have multiple aims*

- **Empowerment**
- **Health literacy**
- **Wellbeing**
- **Self-efficacy**
- **Self-determination**
- **Self-management**
- **Advocacy**
- **Autonomy**
- **Active coping**
- **Shared decision making**
- **Intrinsic motivation**

### Intervention setting

##### Geographical location

- **Country**

##### Setting type

- **Community**
- **Clinical**
- **Online**

*Could also be a combination of settings*

### Child’s primary condition/diagnosis

- **Any long term health condition**
  - **Neurodisability**
    - **Autistic Spectrum Disorder (ASD)**
    - **Epilepsy**
    - **Cerebral palsy (CP)**
    - **Other – condition specific**
    - **Other – generic**
  - **Other – condition specific**
  - **Other – generic**

### Provider

- **Academic Research Team** *– this is anyone based in or affiliated with a HEI.*
- **Service Provider** *– this is anyone who also delivers a service (health or social care) to parent carers.*
- **Parent Carer**
- **Charity or not-for-profit organisation**

*Could also be a combination of providers*

### Type of intervention

- **Parent carer focussed intervention** *– Primary focus on teaching parent carers strategies to improve their own health and/or participation/function.*
- **Parent mediated intervention** *– Primary focus on teaching parents specific parenting skills and strategies they can implement with their children. (given the eligibility criteria, PMIs were only included if there were also clear parent carer focussed outcomes).*
- **Social support Intervention** *– Primary focus on offering access to support structures (based on Social Support Inventory)*
- **Staff training** *– Staff training intervention with clear parent carer focussed main outcomes.*

*Could also be a combination/hybrid type*

### Structure of intervention

*Constructs used to code the structure of interventions were based on the TIDiER Framework.^22^*

##### Mode (How?)

- **Group** – *Intervention delivered to a group*.
- **Individual** – *Intervention delivered to an individual*.
- **Independent** – *Intervention accessed independently*.

##### Platform (How?)

- **Virtual** – *Intervention delivered via an online video communications platform*.
- **Face to face** – *Intervention delivered in person.*
- **Self-paced online** – *Intervention is delivered via an online platform and can be accessed in user’s own time.*

##### Procedure (What?)

- **Information provision (Synchronous or asynchronous)**
- **Interactive activities (Synchronous of asynchronous)**
- **Advocacy work**

##### Materials (What?)

- **Shareable information (physical or online).**
- **Presentations**
- **Intervention Manual**
- **Digital tool**
- **Other training props**
- **None specified**

##### Content (What?)

*The content of the interventions was highly varied, and it was neither practical nor helpful to capture this data on a granular level. The team felt it was feasible to code intervention content using an existing framework based on categories in the online ICF browser.^24^ An additional code ‘Specific content related to child’s health condition’ was also added. Interventions could include content in more than one category.*

- **Self-care** – *content related to caring for oneself and looking after one’s health*.
- **Interpersonal interactions and relationships** – *content related to carrying out the tasks/actions required for basic and complex interactions with others*.
- **Learning and applying knowledge** – *content related to thinking, solving problems and making decisions*.
- **Community, social and civic life** – *content related to carrying our tasks/actions required to engage in organised social life and in community, social and civic areas of life*.
- **Domestic life** – *content related to carrying our domestic and everyday tasks/actions; including care responsibilities for children*.
- **Specific content related to child’s health condition**
- **None specified**

##### Manualised

Was there clear evidence that the intervention was manualised?

- **Yes**
- **No**

### Design Strategy

*There was great variety in the way that interventions were developed and how this was reported. Two elements of intervention design that could be readily be captured were:*

Was there evidence of coproduction with parent carers?

(i.e. were parent carers involved in the development of the intervention).

- **Yes**
- **No**

Was there clear evidence of a published logic model?

- **Yes**
- **No**

### Implementation Strategy

Was there clear evidence of an implementation strategy?

(i.e. a plan to promote the systematic uptake of the intervention into routine practice)*

- **Yes**
- **No**

### Evaluation Strategy

Which study type/approach was used?

- **RCT**
- **RCT (protocol only)**
- **Quasi-experimental study**
- **Pilot**
- **Feasibility**
- **Qualitative**
- **Mixed Methods**
- **None specified**

*Some interventions used multiple approaches.*

##### Outcome measures used

*Outcome measures were coded as each individual measure. Interventions used multiple outcome measures in their evaluations.*

*Definition taken from:

Bauer MS, Damschroder L, Hagedorn H, Smith J, Kilbourne AM. An introduction to implementation science for the non-specialist. BMC psychology. 2015 Dec;3:1-2.
