# Supplementary material for: Interventions supporting the empowerment of parent carers of children with neurodisability and other long‐term health conditions: A scoping review
Source: Dev Med Child Neurol. 2025 Oct 26;68(4):489–500. doi: 10.1111/dmcn.70039 (PMC12982629; doi:10.1111/dmcn.70039)
Supplement: Supplementary file 5 — Appendix S4: Reference list of included information sources. [file DMCN-68-489-s006.docx]

## Appendix S4: Reference list of Included Information Sources.

Acceptance and Commitment therapy program (ASD).

1. Marino F, Failla C, Chilà P, Minutoli R, Puglisi A, Arnao AA, Pignolo L, Presti G, Pergolizzi F, Moderato P, Tartarisco G. The effect of acceptance and commitment therapy for improving psychological well-being in parents of individuals with autism spectrum disorders: a randomized controlled trial. Brain sciences. 2021 Jun 30;11(7):880.

Acceptance and Commitment Therapy Program (Asthma).

1. Chong YY, Mak YW, Leung SP, Lam SY, Loke AY. Acceptance and commitment therapy for parental management of childhood asthma: An RCT. Pediatrics. 2019 Feb 1;143(2).

ACES (Autism, Collaboration, Education, Support).

1. Stravitz SA. Parents as partners: Evaluation of an individualized program that offers autism collaboration, education, and support (ACES) for parents, educators, and clinicians to improve the possibilities of optimal outcomes for children diagnosed with autistic disorder (Doctoral dissertation, Fielding Graduate University).

Advocacy in special education.

1. Andrea Sherwin Ripp. The effects of Internet -based advocacy instruction on the self -determination, psychological empowerment, and self -efficacy of parents of a child with a disability. United States -- New York: Columbia University; 2005.

Advocacy Project.

1. Fazil Q, Wallace LM, Singh G, Ali Z, Bywaters P. Empowerment and advocacy: reflections on action research with Bangladeshi and Pakistani families who have children with severe disabilities. Health & social care in the community. 2004 Sep;12(5):389-97.

AMOR program (Acceptance, Mindfulness, Optimism, Resilience).

1. Schwartzman JM, Millan ME, Uljarevic M, Gengoux GW. Resilience intervention for parents of children with autism: Findings from a randomized controlled trial of the AMOR method. Journal of autism and developmental disorders. 2022 Feb;52(2):738-57.
2. Jessica Schwartzman. Resilience Training for Parents of Children with Autism: Collateral Parent and Child Outcomes. United States -- California: Palo Alto University; 2020.

Asthma Family Empowerment Program (AFEP).

1. Yeh HY, Ma WF, Huang JL, Hsueh KC, Chiang LC. Evaluating the effectiveness of a family empowerment program on family function and pulmonary function of children with asthma: A randomized control trial. International journal of nursing studies. 2016 Aug 1;60:133-44.

Autism 101.

1. Brukilacchio BH. Parent Support Following an Initial Autism Diagnosis: A Mixed Methods Open Trial of Autism 101. The University of Texas at Austin; 2022.

Autism Parent Navigators.

1. Yingling ME, Hock RM, Feinberg ME, Holbert AA. Community-engaged process to adapt evidence-based programs for parents of children with autism spectrum disorder. Children and Youth Services Review. 2020 May 1;112:104876.
2. Hock RM, Rovane AK, Feinberg ME, Jones DE, Holbert AA. A pilot study of a co-parenting intervention for parents of children with autism spectrum disorder. Journal of Child and Family Studies. 2022 Aug;31(8):2091-107.

Autistic Spectrum Condition: Enhancing Nurture and development (ASCEND)

1. Pillay M, Alderson-Day B, Wright B, Williams C, Urwin B. Autism Spectrum Conditions-Enhancing Nurture and Development (ASCEND): An evaluation of intervention support groups for parents. Clinical child psychology and psychiatry. 2011 Jan;16(1):5-20.

Born at the Right Time Courses

1. Born at the right time. Training and Development. [Internet]. UK; 2025 [cited, February 2025]. Available from: <https://www.bornattherighttime.com/training_and_development/>

Bremer Asthma Training for Parents (BASE program).

1. Warschburger P, von Schwerin AD, Buchholz HT, Petermann F. An educational program for parents of asthmatic preschool children: short-and medium-term effects. Patient Education and Counseling. 2003 Sep 1;51(1):83-91.

Bright Coaching.

1. Majnemer A, O'Donnell M, Ogourtsova T, Kasaai B, Ballantyne M, Cohen E, Collet JP, Dewan T, Elsabbagh M, Hanlon-Dearman A, Filliter JH. BRIGHT coaching: a randomized controlled trial on the effectiveness of a developmental coach system to empower families of children with emerging developmental delay. Frontiers in pediatrics. 2019 Aug 7;7:332.

Building on Family Strengths (BFS).

1. Kieckhefer GM, Trahms CM, Churchill SS, Kratz L, Uding N, Villareale N. A randomized clinical trial of the building on family strengths program: an education program for parents of children with chronic health conditions. Maternal and child health journal. 2014 Apr;18:563-74.

CARES intervention.

1. Mills AS, Vimalakanthan K, Sivapalan S, Shanmugalingam N, Weiss JA. Brief report: Preliminary outcomes of a peer counselling program for parents of children with autism in the South Asian community. Journal of Autism and Developmental Disorders. 2021 Jan;51(1):334-40.

Caring for parent caregivers (CPC).

1. Schultz CL, Schultz NC, Bruce EJ, Smyrnios KX, Carey LB, Carey CL. Psychoeducational support for parents of children with intellectual disability: An outcome study. International Journal of Disability, Development and Education. 1993 Jan 1;40(3):205-16.

Caring Parents.

1. Hetzel C, Alles T, Holzer M, Koch E, Froböse I. Does a one-week health program promote well-being among caregiving parents? A quasiexperimental intervention study in Germany. Journal of Public Health. 2022:1-2.

Circle of Security Parenting (COSP).

1. Kubo N, Kitagawa M, Iwamoto S, Kishimoto T. Effects of an attachment-based parent intervention on mothers of children with autism spectrum disorder: preliminary findings from a non-randomized controlled trial. Child and adolescent psychiatry and mental health. 2021 Dec;15:1-4.

Co-facilitated Support Group Intervention.

1. Banach M, Iudice J, Conway L, Couse LJ. Family support and empowerment: Post autism diagnosis support group for parents. Social work with groups. 2010 Jan 12;33(1):69-83.
2. Banach M, Couse LJ. Interdisciplinary co-facilitation of support groups for parents of children with autism: An opportunity for professional preparation. Social Work with Groups. 2012 Oct 1;35(4):313-29.

COMPASS for Hope (C-HOPE)

1. Kuravackel GM, Ruble LA, Reese RJ, Ables AP, Rodgers AD, Toland MD. COMPASS for hope: Evaluating the effectiveness of a parent training and support program for children with ASD. Journal of autism and developmental disorders. 2018 Feb;48:404-16.

Consultation and Collaboration with Families and Colleagues.

1. Murray MM, Handyside LM, Straka LA, Arton-Titus TV. Parent empowerment: connecting with preservice special education teachers. School Community Journal. 2013;23(1):145-68.

Coping Skills training (CST).

1. Haakonsen Smith C, Turbitt E, Muschelli J, Leonard L, Lewis KL, Freedman B, Muratori M, Biesecker BB. Feasibility of coping effectiveness training for caregivers of children with autism spectrum disorder: A genetic counseling intervention. Journal of genetic counseling. 2018 Feb;27:252-62.

Coping Skills training program (CSTP).

1. Gammon EA, Rose SD. The coping skills training program for parents of children with developmental disabilities: An experimental evaluation. Research on Social Work Practice. 1991 Jul;1(3):244-56.

Creating Avenues for Parent Partnership (CAPP).

1. Buelow JM. An intervention for parents of children with epilepsy and significant learning problems: lessons learned from a feasibility study. Journal of the American Psychiatric Nurses Association. 2007 Jun;13(3):146-52.
2. Buelow JM, Johnson CS, Dunn DW, Perkins SM. Satisfaction with "creating avenues for parent partnership;" an intervention for parent's of children with epilepsy. EPILEPSIA 2009 Nov 1 (Vol. 50, pp. 4-4).

Creating Opportunities for Parent Empowerment (COPE).

1. Mazurek Melnyk B, Alpert‐Gillis LJ, Hensel PB, Cable‐Beiling RC, Rubenstein JS. Helping mothers cope with a critically ill child: a pilot test of the COPE intervention. Research in nursing & health. 1997 Feb;20(1):3-14.
2. Duffy LV, Vessey JA. A randomized controlled trial testing the efficacy of the creating opportunities for parent empowerment program for parents of children with epilepsy and other chronic neurological conditions. Journal of Neuroscience Nursing. 2016 Jun 1;48(3):166-74.
3. Sheijani MJ, Chehrzad MM, Masouleh SR, Leyli EK, Bidabadi E. The effect of “Creating opportunities for parent empowerment” program on parents of children with Epilepsy and other chronic neurological conditions. Iranian Journal of Child Neurology. 2020;14(1):65.
4. Peek G, Melnyk BM. A coping intervention for mothers of children diagnosed with cancer: Connecting theory and research. Applied Nursing Research. 2014 Aug 1;27(3):202-4.

Cygnet.

1. Stuttard L, Beresford B, Clarke S, Beecham J, Morris A. An evaluation of the Cygnet parenting support programme for parents of children with autism spectrum conditions. Research in Autism Spectrum Disorders. 2016 Mar 1;23:166-78.

Digital Micro Attachment Intervention.

1. Vandesande S, Bosmans G, Sterkenburg P, Schuengel C, Maes B. Piloting attachment psychoeducation provided to parents of children with severe disabilities: Testing the feasibility of a digital micro-intervention. Journal of Intellectual Disabilities. 2023 Jun;27(2):433-50.

e-Powered Parents.

1. Geense WW, van Gaal BG, Knoll JL, Cornelissen EA, Schoonhoven L, Kok G. Online support program for parents of children with a chronic kidney disease using intervention mapping: a development and evaluation protocol. JMIR research protocols. 2016 Jan 13;5(1):e4837.
2. Geense WW, van Gaal BG, Knoll JL, Maas NM, Kok G, Cornelissen EA, Nijhuis-van der Sanden MW. Effect and process evaluation of e-powered parents, a web-based support program for parents of children with a chronic kidney disease: feasibility randomized controlled trial. Journal of medical Internet research. 2018 Aug 1;20(8):e9547.

Early Bird and Early Bird Plus.

1. Shields J. The NAS EarlyBird Programme: Partnership with parents in early intervention. Autism. 2001 Mar;5(1):49-56.
2. Palmer M, San José Cáceres A, Tarver J, Howlin P, Slonims V, Pellicano E, Charman T. Feasibility study of the National Autistic Society EarlyBird parent support programme. Autism. 2020 Jan;24(1):147-59.
3. Dawson-Squibb JJ, Davids EL, de Vries PJ. Scoping the evidence for EarlyBird and EarlyBird Plus, two United Kingdom-developed parent education training programmes for autism spectrum disorder. Autism. 2019 Apr;23(3):542-55.

Early intervention readiness program (EIRP).

1. Tolmie RS, Bruck S, Kerslake R. The early intervention readiness program (EIRP) A post-ASD diagnosis family support program. Topics in Early Childhood Special Education. 2017 Feb;36(4):242-50.

Early Positive Approaches to Support (EPAtS).

1. Coulman E, Gore N, Moody G, Wright M, Segrott J, Gillespie D, Petrou S, Lugg-Widger F, Kim S, Bradshaw J, McNamara R. Early positive approaches to support (E-PatS) for families of young children with intellectual disability: A feasibility randomised controlled trial. Frontiers in Psychiatry. 2021 Dec 21;12:729129.

EMPOWER - Autism.

1. Leadbitter, K., Smallman, R., James, K., Shields, G., Ellis, C., Langhorne, S., Harrison, L., Hackett, L., Dunkerley, A., Kroll, L. and Davies, L., 2022. REACH-ASD: a UK randomised controlled trial of a new post-diagnostic psycho-education and acceptance and commitment therapy programme against treatment-as-usual for improving the mental health and adjustment of caregivers of children recently diagnosed with autism spectrum disorder. *Trials*, *23*(1), p.585.

Empowered Parents Program.

1. Ned DM. *Expert Review of the Empowered Parents Program: A Psychoeducation-Support Group* (Doctoral dissertation, The Chicago School of Professional Psychology).

Empowering Education Program.

1. McCarthy MJ, Herbert R, Brimacombe M, Hansen J. Empowering parents through asthma education. Pediatric Nursing. 2002 Sep 1;28(5):465.

Empowering Families Curriculum.

1. Blair ME. Evaluation of three parent-focused disability information and healthy lifestyle curriculum modules for Latino parents of children with developmental disabilities. Utah State University; 2008.

Empowering Families Program. DESSA.

1. DESSA (Disability Equality Specialist Support Agency). Empowering Families Program. [Internet]. Ireland; 2025 [cited February, 2025]. Available from: <https://www.dessa.ie/training/empowering-parents/>

Empowering grandparents program.

1. McCallion P, Janicki MP, Grant-Griffin L, Kolomer S. Grandparent carers II: Service needs and service provision issues. InGrandparents as Carers of Children with Disabilities 2014 Mar 18 (pp. 57-84). Routledge.
2. McCallion P, Janicki MP, Kolomer SR. Controlled evaluation of support groups for grandparent caregivers of children with developmental disabilities and delays. American Journal on Mental Retardation. 2004 Sep 1;109(5):352-61.

Empowering Unpaid Parent Carers for Positive Change. Refresh Carers.

1. Refresh Carers. Empowering Unpaid Parent Carers for Positive Change. [Internet]. UK; 2025. [cited February, 2025]. Available from: <https://www.refreshcarers.com/>

ENabling VISions And Growing Expectations (ENVISAGE).

1. Miller L, Nickson G, Pozniak K, Khan D, Imms C, Ziviani J, Cross A, Martens R, Cavalieros V, Rosenbaum P. ENabling VISions and Growing Expectations (ENVISAGE): Parent reviewers’ perspectives of a co-designed program to support parents raising a child with an early-onset neurodevelopmental disability. Research in Developmental Disabilities. 2021 Dec 20;121:1-4.
2. Miller L, Imms C, Cross A, Pozniak K, O’Connor B, Martens R, Cavalieros V, Babic R, Novak-Pavlic M, Rodrigues M, Balram A. Impact of “early intervention” parent workshops on outcomes for caregivers of children with neurodisabilities: a mixed-methods study. Disability and rehabilitation. 2023 Nov 6;45(23):3900-11.
3. Pozniak K, Cross A, Babic R, Cavalieros V, Martens R, Rosenbaum P, Imms C, Novak‐Pavlic M, Balram A, Hughes D, O'Connor B. Co‐development of the ENVISAGE‐families programme for parents of children with disabilities: Reflections on a parent–researcher partnership. Australian Occupational Therapy Journal. 2022 Dec;69(6):653-61.

ENHANCE program.

1. Whiting M, Nash AS, Kendall S, Roberts SA. Enhancing resilience and self-efficacy in the parents of children with disabilities and complex health needs. Primary Health Care Research & Development. 2019 Jan;20:e33.

Evidence Based Practice program.

1. Murphy A, Trembath D, Arciuli J, Roberts JM. Supporting parents of children with autism spectrum disorders to become informed consumers of evidence on speech pathology practice. Evidence-Based Communication Assessment and Intervention. 2011 Jun 1;5(2):116-29.

Face 2 Face Program.

1. Bray L, Carter B, Sanders C, Blake L, Keegan K. Parent-to-parent peer support for parents of children with a disability: A mixed method study. Patient Education and Counseling. 2017 Aug 1;100(8):1537-43.
2. Blake L, Bray L, Carter B. “It’sa lifeline”: Generating a sense of social connectedness through befriending parents of disabled children or children with additional need. Patient Education and Counseling. 2019 Dec 1;102(12):2279-85.

FACES (Fostering Advocacy, Communication, Empowerment and Support).

1. Pearson JN, Meadan H. FACES: An advocacy intervention for African American parents of children with autism. Intellectual and Developmental Disabilities. 2021 Apr 1;59(2):155-71.

Families Linking with Families (FLWF).

1. Gibbs A, Flanagan J, Gray L. An Australian online training and support program for caregivers of children and youth with Fetal Alcohol Spectrum Disorder: Families linking with families. Journal of intellectual & developmental disability. 2024 Apr 2;49(2):175-85.
2. Gibbs A, Harrington S, Robinson C, Brooks C, Dedman C. Getting on With It”: a course by caregivers, for caregivers—pilot overview and evaluation report. NOFASD Australia. 2020 Mar 23.
3. Gibbs A. An evidence-based training and support course for caregivers of children with foetal alcohol spectrum disorder (FASD) in New Zealand. Advances in dual diagnosis. 2019 Feb 28;12(1/2):73-84.

Family Centred Early Intervention Program.

1. Muthukaruppan SS, Cameron C, Campbell Z, Krishna D, Moineddin R, Bharathwaj A, Poomariappan BM, Mariappan S, Boychuk N, Ponnusamy R, MacLachlan J. Impact of a family-centred early intervention programme in South India on caregivers of children with developmental delays. Disability and rehabilitation. 2022 May 22;44(11):2410-9.

Family Centred Empowement Model. Gholami (2019)

1. Gholami S, Besharati R, Haresabadi M, Ghorbanzadeh M, Sarani A, Hamedi A. The Impact of Family-Centered Empowerment Program on the Quality of Life of Mothers with Epileptic Children. Journal of Kerman University of Medical Sciences. 2019 Sep 1;26(5):349-56.

Family Centred Empowement Model. Rostaminasab (2023)

1. Rostaminasab S, Nematollahi M, Jahani Y, Mehdipour-Rabori R. The effect of family-centered empowerment model on burden of care in parents and blood glucose level of children with type I diabetes family empowerment on burden of care and HbA1C. BMC nursing. 2023 Jun 20;22(1):214.

Family Centred Empowement Model. Shoghi (2019)

1. Shoghi M, Shahbazi B, Seyedfatemi N. The effect of the Family-Centered Empowerment Model (FCEM) on the care burden of the parents of children diagnosed with cancer. Asian Pacific journal of cancer prevention: APJCP. 2019;20(6):1757.

Family Centred Empowerment Model. Boshagh (2022)

1. Boshagh F, Hakim A, Alghasi A, Haghighizadeh MH. Effect of family-centered empowerment model on knowledge and caring performance of mothers of children with leukemia: a randomized clinical trial. Jundishapur J Chronic Dis Care. 2022;11(3):1-7.

Family Centred Empowerment Model. Ghaljaei (2022)

1. Ghaljaei F, Motamedi M, Saberi N, ArbabiSarjou A. The effect of the family-centered empowerment model on family functioning in type 1 diabetic children: a quasi-experimental study. Medical-Surgical Nursing Journal. 2022 Jan 1;11(2).

Family Centred Empowerment Model. Rahgoi (2019)

1. Rahgoi A, Sojoodi T, Khoshknab MF, Rahgozar M, Shahshahani S. Effects of empowerment program on the burden of care in mothers of children with phenylketonuria. Iranian journal of child neurology. 2019;13(2):53.

Family Centred Short Course.

1. Samadi SA, McConkey R, Kelly G. Enhancing parental well-being and coping through a family-centred short course for Iranian parents of children with an autism spectrum disorder. Autism. 2013 Jan;17(1):27-43.

Family Empowerment Program.

1. Kashaninia Z, Payrovee Z, Soltani R, Mahdaviani SA. Effect of family empowerment on asthma control in school-age children. Tanaffos. 2018 Jan;17(1):47.

Family Focussed pyschoeducation therapy (FFPT).

1. Zhou Y, Yin H, Wang M, Wang J. The effect of family-focused psychoeducational therapy for autism spectrum disorder children's parents on parenting self-efficacy and emotion. Archives of psychiatric Nursing. 2019 Feb 1;33(1):17-22.

Family implemented TEACCH for toddlers (FITT).

1. Turner-Brown L, Hume K, Boyd BA, Kainz K. Preliminary efficacy of family implemented TEACCH for toddlers: Effects on parents and their toddlers with autism spectrum disorder. Journal of autism and developmental disorders. 2019 Jul 15;49:2685-98.

Family Partners Program.

1. Pollock MD, Ming D, Chung RJ, Maslow G. Parent-to-parent peer support for children and youth with special health care needs: Preliminary evaluation of a family partner program in a healthcare system. Journal of Pediatric Nursing. 2022 Sep 1;66:6-14.

Family strengths orientated therapeutic conversations (FAM-SOTC)

1. Svavarsdottir EK, Kamban SW, Konradsdottir E, Sigurdardottir AO. The impact of family strengths oriented therapeutic conversations on parents of children with a new chronic illness diagnosis. Journal of Family Nursing. 2020 Aug;26(3):269-81.

Family Support Hub. Together for Short Lives.

1. Together for short lives. Family Support Hub. [Internet]. UK; 2025 [cited, February 2025]. Available from: <https://www.togetherforshortlives.org.uk/get-support/>

Family-Professional Collaboration Model.

1. An M, Palisano RJ. Family–professional collaboration in pediatric rehabilitation: A practice model. Disability and rehabilitation. 2014 Mar 1;36(5):434-40.
2. An M, Palisano RJ, Yi CH, Chiarello LA, Dunst CJ, Gracely EJ. Effects of a collaborative intervention process on parent empowerment and child performance: A randomized controlled trial. Physical & Occupational Therapy in Pediatrics. 2019 Jan 2;39(1):1-5.

FAMOSES.

1. Wohlrab GC, Rinnert S, Bettendorf U, Fischbach H, Heinen G, Klein P, Kluger G, Jacob K, Rahn D, Winter R, Pfäfflin M. Famoses: a modular educational program for children with epilepsy and their parents. Epilepsy & Behavior. 2007 Feb 1;10(1):44-8.
2. Pfäfflin M, Petermann F, Rau J, May TW. The psychoeducational program for children with epilepsy and their parents (FAMOSES): results of a controlled pilot study and a survey of parent satisfaction over a five-year period. Epilepsy & Behavior. 2012 Sep 1;25(1):11-6.
3. Hagemann A, Pfäfflin M, Nussbeck FW, May TW. The efficacy of an educational program for parents of children with epilepsy (FAMOSES): Results of a controlled multicenter evaluation study. Epilepsy & Behavior. 2016 Nov 1;64:143-51.

FIRME (Familias Incluidas en Recibiendo Mejor Education Especial).

1. Rios K. *A study of the families included in receiving better special education services project for Latino families of children with disabilities* (Doctoral dissertation, University of Illinois at Urbana-Champaign).

Good Googling.

1. Armstrong-Heimsoth A, Johnson ML, McCulley A, Basinger M, Maki K, Davison D. Good Googling: a consumer health literacy program empowering parents to find quality health information online. Journal of Consumer Health on the Internet. 2017 Apr 3;21(2):111-24.

Gratitude Intervention.

1. Timmons L, Ekas NV. Giving thanks: Findings from a gratitude intervention with mothers of children with autism spectrum disorder. Research in Autism Spectrum Disorders. 2018 May 1;49:13-24.

Health Promotion Tool Kit.

1. Ghoneim AA. Health promotion toolkit: An approach for empowering families caring for children with developmental disabilities in Tabuk. Open Access Macedonian Journal of Medical Sciences. 2018 Aug 5;6(8):1503.

Healthly living Triple-P.

1. Mitchell AE, Morawska A, Lohan A, Filus A, Batch J. Randomised controlled trial of the Healthy Living Triple P–Positive Parenting Program for families of children with type 1 diabetes. Journal of Child Health Care. 2024 Jun;28(2):235-55.
2. Morawska A, Mitchell AE, Burgess S, Fraser J. Fathers’ perceptions of change following parenting intervention: Randomized controlled trial of Triple P for parents of children with asthma or eczema. Journal of Pediatric Psychology. 2017 Aug 1;42(7):792-803.
3. Morawska A, Mitchell A, Burgess S, Fraser J. Randomized controlled trial of Triple P for parents of children with asthma or eczema: Effects on parenting and child behavior. Journal of Consulting and Clinical Psychology. 2017 Apr;85(4):283.
4. Mitchell AE, Morawska A, Kirby G, McGill J, Coman D, Inwood A. Triple P for parents of children with Phenylketonuria: a Nonrandomized Trial. Journal of pediatric psychology. 2021 Mar 1;46(2):208-18.

Healthy Mothers Healthy Families (HMHF).

1. Bourke-Taylor HM, Jane FM. Mothers’ experiences of a women’s health and empowerment program for mothers of a child with a disability. Journal of autism and developmental disorders. 2018 Jun;48:2174-86.
2. Harris V, Bourke‐Taylor HM, Leo M. Healthy Mothers Healthy Families, Health Promoting Activity Coaching for mothers of children with a disability: Exploring mothers' perspectives of programme feasibility. Australian Occupational Therapy Journal. 2022 Dec;69(6):662-75.
3. Bourke-Taylor HM, Joyce KS, Grzegorczyn S, Tirlea L. Mental health and health behaviour changes for mothers of children with a disability: Effectiveness of a health and wellbeing workshop. Journal of Autism and Developmental Disorders. 2022 Feb;52(2):508-21.
4. Bourke‐Taylor HM, Grzegorczyn S, Joyce KS. Peer mentor training: Pathway to competency for facilitators of Healthy Mothers Healthy Families workshops. Child: Care, Health and Development. 2021 Sep;47(5):575-87.
5. Bourke-Taylor HM, Jane F, Peat J. Healthy mothers healthy families workshop intervention: A preliminary investigation of healthy lifestyle changes for mothers of a child with a disability. Journal of autism and developmental disorders. 2019 Mar 15;49:935-49.

Healthy Parent Carers (HPC).

1. Borek AJ, McDonald B, Fredlund M, Bjornstad G, Logan S, Morris C. Healthy Parent Carers programme: development and feasibility of a novel group-based health-promotion intervention. BMC public health. 2018 Dec;18:1-6.
2. Lloyd J, Bjornstad G, Borek A, Cuffe-Fuller B, Fredlund M, McDonald A, Tarrant M, Berry V, Wilkinson K, Mitchell S, Gillett A. Healthy Parent Carers programme: mixed methods process evaluation and refinement of a health promotion intervention. BMJ open. 2021 Aug 1;11(8):e045570.
3. Garrood A, Bjornstad G, Borek A, Gillett A, Lloyd J, Brand S, Tarrant M, Ball S, Hawton A, McDonald A, Fredlund M. Healthy Parent Carers: Acceptability and practicability of online delivery and learning through implementation by delivery partner organisations. Health Expectations. 2023 Oct;26(5):2050-63.
4. Bjornstad G, Cuffe-Fuller B, Ukoumunne OC, Fredlund M, McDonald A, Wilkinson K, Lloyd J, Hawton A, Berry V, Tarrant M, Borek A. Healthy Parent Carers: feasibility randomised controlled trial of a peer-led group-based health promotion intervention for parent carers of disabled children. Pilot and feasibility studies. 2021 Dec;7:1-8.

HOPE Intervention.

1. Martin F, Clyne W, Pearce G, Turner A. Self-management support intervention for parents of children with developmental disorders: The role of gratitude and hope. Journal of Child and Family Studies. 2019 Apr 1;28:980-92.
2. Hall KR. HOPE for Parents of Children with an Autism Spectrum Disorder. Hofstra University; 2021.

Incredible Years Program.

1. Dababnah S, Parish SL. Feasibility of an empirically based program for parents of preschoolers with autism spectrum disorder. Autism. 2016 Jan;20(1):85-95.
2. Dababneh S, Parish SL. Incredible years program tailored to parents of preschoolers with autism: Pilot results. Research on Social Work Practice. 2016 Jul;26(4):372-85.

Intensive family competence intervention.

1. Dellve L, Samuelsson L, Tallborn A, Fasth A, Hallberg LR. Stress and well‐being among parents of children with rare diseases: A prospective intervention study. Journal of advanced nursing. 2006 Feb;53(4):392-402.

I - InTERACT

1. Raj SP, Shultz EL, Zang H, Zhang N, Kirkwood MW, Taylor HG, Stancin T, Yeates KO, Wade SL. Effects of web-based parent training on caregiver functioning following pediatric traumatic brain injury: a randomized control trial. The Journal of Head Trauma Rehabilitation. 2018 Nov 1;33(6):E19-29.
2. Antonini TN, Raj SP, Oberjohn KS, Wade SL. An online positive parenting skills programme for paediatric traumatic brain injury: Feasibility and parental satisfaction. Journal of telemedicine and telecare. 2012 Sep;18(6):333-8.
3. Antonini TN, Raj SP, Oberjohn KS, Cassedy A, Makoroff KL, Fouladi M, Wade SL. A pilot randomized trial of an online parenting skills program for pediatric traumatic brain injury: improvements in parenting and child behavior. Behavior therapy. 2014 Jul 1;45(4):455-68.

Just ADD Mums.

1. Home A, Biggs T. Evidence-based practice in the real world. Groupwork. 2005;15(2):39-60.

Keeping it Together (KIT).

1. Stewart D, Law M, Burke‐Gaffney J, Missiuna C, Rosenbaum P, King G, Moning T, King S. Keeping it togetherTM: an information KIT for parents of children and youth with special needs. Child: Care, Health and Development. 2006 Jul;32(4):493-500.

Life skills training.

1. Kirkham MA, Schilling RF. Life skills training with mothers of handicapped children. InAdvances in Group Work Research 2018 Oct 24 (pp. 67-88). Routledge.

Maximising Adolescent Post-secondary success (MAPSS).

1. Kirby AV, Feldman KJ, Himle MB, Diener ML, Wright CA, Hoffman JM. Pilot test of the Maximizing Adolescent Post-Secondary Success (MAPSS) intervention: Supporting parents of autistic youth. The American Journal of Occupational Therapy. 2021 May 1;75(3).

MePrEPA (metas, preguntor, escuchar, pregnator, para aclarar).

1. Thomas KC, Stein GL, Williams CS, Jolles MP, Sleath BL, Martinez M, García SJ, Guzman LE, Williams CE, Morrissey JP. Fostering activation among Latino parents of children with mental health needs: An RCT. Psychiatric Services. 2017 Oct 1;68(10):1068-75.

Mind the Gap

1. Iadarola S, Pellecchia M, Stahmer A, Lee HS, Hauptman L, Hassrick EM, Crabbe S, Vejnoska S, Morgan E, Nuske H, Luelmo P. Mind the gap: An intervention to support caregivers with a new autism spectrum disorder diagnosis is feasible and acceptable. Pilot and feasibility studies. 2020 Dec;6:1-3.

Multi-disciplinary Parent Education Program.

1. Ji B, Sun M, Yi R, Tang S. Multidisciplinary parent education for caregivers of children with autism spectrum disorders. Archives of Psychiatric Nursing. 2014 Oct 1;28(5):319-26.

MyAsthma.

1. Fiks AG, Mayne S, Karavite DJ, DeBartolo E, Grundmeier RW. A shared e-decision support portal for pediatric asthma. The Journal of ambulatory care management. 2014 Apr 1;37(2):120-6.
2. Fiks AG, Mayne SL, Karavite DJ, Suh A, O’Hara R, Localio AR, Ross M, Grundmeier RW. Parent-reported outcomes of a shared decision-making portal in asthma: a practice-based RCT. Pediatrics. 2015 Apr 1;135(4):e965-73.

MyQuality - Website.

1. Harris N, Beringer A, Fletcher M. Families’ priorities in life-limiting illness: improving quality with online empowerment. Archives of disease in childhood. 2016 Mar 1;101(3):247-52.
2. Harris N. eHealth and the delivery of person-centred care for children with life-limiting conditions: a realist evaluation. University of the West of England; 2022 Oct.

New Bold Hope

1. New Bold Hope. Family Toolbox. [Internet]. UK;2025. [cited February 2025]. Available from: https://www.newboldhope.com/family-toolbox-general-information

Nurturing Families - Special Needs and Health Challenges Curriculum.

1. Burton RS, Zwahr-Castro J, Magrane CL, Hernandez H, Farley LG, Amodei N. The nurturing program: An intervention for parents of children with special needs. Journal of child and family studies. 2018 Apr;27:1137-49.

Ohana Project.

1. Wilford JG, McCarty R, Torno L, Mucci G, Torres-Eaton N, Shen V, Loudon W. A multi-modal family peer support-based program to improve quality of life among pediatric brain tumor patients: a mixed-methods pilot study. Children. 2020 Apr 20;7(4):35.

Online Parent Information and Support (OPIS).

1. Swallow VM, Knafl K, Santacroce S, Campbell M, Hall AG, Smith T, Carolan I. An interactive health communication application for supporting parents managing childhood long-term conditions: outcomes of a randomized controlled feasibility trial. JMIR research protocols. 2014 Dec 3;3(4):e3716.

Online Parent Support Group Intervention.

1. Martin S, Roderick MC, Lockridge R, Toledo-Tamula MA, Baldwin A, Knight P, Wolters P. Feasibility and preliminary efficacy of an Internet support group for parents of a child with neurofibromatosis type 1: a pilot study. Journal of genetic counseling. 2017 Jun;26:576-85.

Online Self Compassion Intervention.

1. Ahmed AN, Raj SP. Self-compassion intervention for parents of children with developmental disabilities: A feasibility study. Advances in neurodevelopmental disorders. 2023 Jun;7(2):277-89.

Parent Child Training Program (PCTP)

1. Brezis RS, Weisner TS, Daley TC, Singhal N, Barua M, Chollera SP. Parenting a child with autism in India: Narratives before and after a parent–child intervention program. Culture, Medicine, and Psychiatry. 2015 Jun;39:277-98.

Parent Empowerment Program (PEP).

1. Rodriguez J, Olin SS, Hoagwood KE, Shen S, Burton G, Radigan M, Jensen PS. The development and evaluation of a parent empowerment program for family peer advocates. Journal of Child and Family Studies. 2011 Aug;20:397-405.
2. Olin SS, Hoagwood KE, Rodriguez J, Ramos B, Burton G, Penn M, Crowe M, Radigan M, Jensen PS. The application of behavior change theory to family-based services: Improving parent empowerment in children’s mental health. Journal of child and family studies. 2010 Aug;19:462-70.
3. Jamison JM, Fourie E, Siper PM, Trelles MP, George-Jones J, Buxbaum Grice A, Krata J, Holl E, Shaoul J, Hernandez B, Mitchell L. Examining the efficacy of a family peer advocate model for Black and Hispanic caregivers of children with autism spectrum disorder. Journal of autism and developmental disorders. 2017 May;47:1314-22.
4. The Reach Institute. Parent Empowerment Program. [Internet]. UK;2025. [cited February 2025]. Available from : <https://thereachinstitute.org/course/parent-empowerment-program-pep/>

Parent Empowerment Program for Asthma Care (PEPAC).

1. Horn IB, Mitchell SJ, Gillespie CW, Burke KM, Godoy L, Teach SJ. Randomized trial of a health communication intervention for parents of children with asthma. Journal of Asthma. 2014 Nov 1;51(9):989-95.

Parent IEP Advocacy Program.

1. Luelmo P, Kasari C, Fiesta Educativa, Inc. Randomized pilot study of a special education advocacy program for Latinx/minority parents of children with autism spectrum disorder. Autism. 2021 Aug;25(6):1809-15.

Parent Management Training (PMT).

1. Sofronoff K, Farbotko M. The effectiveness of parent management training to increase self-efficacy in parents of children with Asperger syndrome. Autism. 2002 Sep;6(3):271-86.

Parent Plus - Special needs.

1. McMahon SM, Wilson CE, Sharry J. Parents Plus parenting programme for parents of adolescents with intellectual disabilities: A cluster randomised controlled trial. Journal of Applied Research in Intellectual Disabilities. 2023 Jul;36(4):871-80.

Parent Support Group Intervention. Bunning (2020)

1. Bunning K, Gona JK, Newton CR, Andrews F, Blazey C, Ruddock H, Henery J, Hartley S. Empowering self-help groups for caregivers of children with disabilities in Kilifi, Kenya: Impacts and their underlying mechanisms. PLoS One. 2020 Mar 9;15(3):e0229851
2. Bunning K, Gona JK, Newton CR, Hartley S. Empowering caregivers of children with learning and developmental disabilities: from situation analysis to community-based inclusive development in Kilifi, Kenya. Tizard Learning Disability Review. 2022 Mar 8;27(1):1-0.

Parent Support Group Intervention. Wakimizu (2022)

1. Wakimizu R, Fujioka H, Nishigaki K, Sato I, Iwata N, Matsuzawa A. Development of family empowerment programs for caregivers of children with disabilities at home: Interim report up to" implementation of pretesting". Journal of International Nursing Research. 2022 Feb 24;1(1):e2021-0004.
2. Wakimizu R, Matsuzawa A, Fujioka H, Nishigaki K, Sato I, Suzuki S, Iwata N. Effectiveness of a peer group-based online intervention program in empowering families of children with disabilities at home. Frontiers in pediatrics. 2022 Oct 24;10:929146.

Parent Support Group Intervention. Adesida (1999)

1. Adesida O, Foreman D. A support group for parents of children with hyperkinetic disorder: an empowerment model. Clinical Child Psychology and Psychiatry. 1999 Oct;4(4):567-78.

Parent to Parent Program. Singer (1999)

1. Singer GS, Marquis J, Powers LK, Blanchard L, Divenere N, Santelli B, Ainbinder JG, Sharp M. A multi-site evaluation of parent to parent programs for parents of children with disabilities. Journal of early Intervention. 1999 Jul;22(3):217-29.

Parent to Parent Program. Wood (2016)

1. Wood LB. Parent-to-parent peer support for diverse low-income families of children with disabilities: A qualitative interview study of a self-help program as part of a medical home model. University of California, Santa Barbara; 2016.

Parent Training Program. Al-Khalaf (2014)

1. Al-Khalaf A, Dempsey I, Dally K. The effect of an education program for mothers of children with autism spectrum disorder in Jordan. International Journal for the Advancement of Counselling. 2014 Jun;36:175-87.

Parent Training Program. Chiang (2014)

1. Chiang HM. A parent education program for parents of Chinese American children with autism spectrum disorders (ASDs) a pilot study. Focus on Autism and Other Developmental Disabilities. 2014 Jun;29(2):88-94.

Parent Training Program. Eo (2005)

1. Eo YS. Effects of an empowerment program on the burden of mothers having a child with cerebral palsy. Journal of Korean Academy of Nursing. 2005 Feb 1;35(1):154-64.

Parent Training Program. Grenier-Martin (2022)

1. Grenier-Martin J, Rivard M, Patel S, Lanovaz MJ, Lefebvre C. Randomized controlled trial on an online training to support caregivers of young children with intellectual and developmental disability managing problem behaviors at home. Journal of Child and Family Studies. 2022 Dec;31(12):3485-97.

Parent Training Program. Krishnan (2018)

1. Krishnan R, Ram D, Hridya VM, Santhosh AJ. Effectiveness of psychoeducation on psychological wellbeing and self-determination in key caregivers of children with intellectual disability. Indian Journal of Psychiatric Social Work. 2018 Mar 1:4-11.

Parent Training Program. Kroodsma (2008)

1. Kroodsma L. An educational workshop for parents of children with Asperger syndrome. Antioch University New England; 2008.

Parent Training Program. McAleese (2014)

1. McAleese A, Lavery C, Dyer KF. Evaluating a psychoeducational, therapeutic group for parents of children with autism spectrum disorder. Child Care in Practice. 2014 Apr 3;20(2):162-81.

Parent Training Program. Milgramm (2022)

1. Milgramm A, Corona LL, Janicki-Menzie C, Christodulu KV. Community-based parent education for caregivers of children newly diagnosed with autism spectrum disorder. Journal of autism and developmental disorders. 2022 Mar;52(3):1200-10.

Parent Training Program. Patra (2015)

1. Patra S, Arun P, Chavan BS. Impact of psychoeducation intervention module on parents of children with autism spectrum disorders: A preliminary study. Journal of neurosciences in rural practice. 2015 Oct;6(4):529.

Parent Training Program. Picard (2014)

1. Picard I, Morin D, De Mondehare L. Psychoeducational program for parents of adolescents with intellectual disabilities. Journal of Policy and Practice in Intellectual Disabilities. 2014 Dec;11(4):279-92.

Parent Training Program. Pilon (1985)

1. Pilon BH, Smith KA. A parent group for the Hispanic parents of children with severe cerebral palsy. Children's Health Care. 1985 Sep 1;14(2):96-102.

Parent Training Program. Shahin (2021)

1. Shahin MA, Hussien RM. Knowledge, attitude, practice, and self-efficacy of caregivers of children with epilepsy: impact of a structured educational intervention program. Epilepsy & Seizure. 2021;13(1):1-6.

Parental Wellbeing Program. The Behaviour Support Hub.

1. The Behaviour Support Hub. Parent Wellbeing Program. [Internet]. UK;2025. [cited February, 2025]. Available from: <https://behavioursupporthub.org.uk/programme/parental-wellbeing-programme/?target=parents>

Parents Taking Action (PTA).

1. Magaña S, Lopez K, Machalicek W. Parents taking action: A psycho‐educational intervention for Latino parents of children with autism spectrum disorder. Family process. 2017 Mar;56(1):59-74.
2. Zeng W, Magaña S, Lopez K, Xu Y, Marroquín JM. Revisiting an RCT study of a parent education program for Latinx parents in the United States: Are treatment effects maintained over time?. Autism. 2022 Feb;26(2):499-512.
3. Magana S, Tejero Hughes M, Salkas K, Gonzales W, Núñez G, Morales M, Garcia Torres M, Moreno-Angarita M. Implementing a parent education intervention in Colombia: Assessing parent outcomes and perceptions across delivery modes. Focus on Autism and Other Developmental Disabilities. 2021 Sep;36(3):165-75.
4. Magaña S, Lopez K, Salkas K, Iland E, Morales MA, Garcia Torres M, Zeng W, Machalicek W. A randomized waitlist-control group study of a culturally tailored parent education intervention for Latino parents of children with ASD. Journal of autism and developmental disorders. 2020 Jan;50:250-62.
5. Torres MG. *Evaluation of the program parents taking action for parents of preadolescents with autism in Colombia* (Doctoral dissertation, University of Illinois at Chicago).

Paediatric and Adolescent support service (PASS) model.

1. Morison JE, Bromfield LM, Cameron HJ. A therapeutic model for supporting families of children with a chronic illness or disability. Child and Adolescent Mental Health. 2003 Sep;8(3):125-30.

Pivotal Response Training (PRT) Group Program.

1. Minjarez MB, Williams SE, Mercier EM, Hardan AY. Pivotal response group treatment program for parents of children with autism. Journal of autism and developmental disorders. 2011 Jan;41:92-101.
2. Verschuur R, Huskens B, Didden R. Effectiveness of parent education in pivotal response treatment on pivotal and collateral responses. Journal of autism and developmental disorders. 2019 Sep 15;49:3477-93.
3. Minjarez MB, Mercier EM, Williams SE, Hardan AY. Impact of pivotal response training group therapy on stress and empowerment in parents of children with autism. Journal of Positive Behavior Interventions. 2013 Apr;15(2):71-8.

Positive Alternatives for Families (PAF).

1. Farber ML, Maharaj R. Empowering high-risk families of children with disabilities. Research on social work practice. 2005 Nov;15(6):501-15.

Positive Futures Project.

1. McConkey R, O’Hagan P, Corcoran J. The impact of a family-centred intervention for parents of children with developmental disabilities: A model project in rural Ireland. Children. 2023 Jan 17;10(2):175.

Programme d'intervention Familiale (PRIFAM).

1. Pelchat D, Lefebvre H. A holistic intervention programme for families with a child with a disability. Journal of Advanced Nursing. 2004 Oct;48(2):124-31.
2. Pelchat D, Lefebvre H, Damiani C. Grief: Adaptation of skills: Transformation: The way in which the Family Intervention Program PRIFAM supports the families of disabled children. Pratiques Psychologiques. 2002;1:41-52.

Pursuit of Wellbeing program.

1. Young D, Reynolds J, Tonmukayakul U, Carter R, Swift E, Williams K, McDonald R, Reddihough D, Carracher R, Ireland P, Tracy J. An intervention to improve the self-efficacy of key workers to support parental wellbeing at an early childhood intervention service in Australia: a stepped wedged randomized cluster trial. Disability and Rehabilitation. 2023 Sep 11;45(19):3046-58.
2. Davis E, Young D, Gilson KM, Reynolds J, Carter R, Tonmukayakul U, Williams K, Gibbs L, McDonald R, Reddihough D, Tracy J. A capacity building program to improve the Self-Efficacy of key workers to support the Well-Being of parents of a child with a disability accessing an early childhood intervention service: Protocol for a Stepped-Wedge design trial. JMIR research protocols. 2019 Apr 3;8(4):e12531.

Resilience Training Program.

1. Luo Y, Xia W, Cheung AT, Ho LL, Zhang J, Xie J, Xiao P, Li HC. Effectiveness of a mobile device–based resilience training program in reducing depressive symptoms and enhancing resilience and quality of life in parents of children with cancer: randomized controlled trial. Journal of medical Internet research. 2021 Nov 29;23(11):e27639.
2. Luo Y, Li HC, Cheung AT, Ho LL, Xia W, Zhang J. Evaluating the experiences of parents of children with cancer engaging in a mobile device-based resilience training programme: a qualitative study. Supportive Care in Cancer. 2022 Jul;30(7):6205-14.

Resourceful Adolescent Parent Program (RAP-P-ASD).

1. Shochet IM, Saggers BR, Carrington SB, Orr JA, Wurfl AM, Duncan BM. A strength-focused parenting intervention may be a valuable augmentation to a depression prevention focus for adolescents with autism. Journal of Autism and Developmental Disorders. 2019 May 15;49:2080-100.

Riding the Rapids - Living with Autism or Disability.

1. Todd S, Bromley J, Ioannou K, Harrison J, Mellor C, Taylor E, Crabtree E. Using group‐based parent training interventions with parents of children with disabilities: A description of process, content and outcomes in clinical practice. Child and Adolescent Mental Health. 2010 Sep;15(3):171-5.
2. Stuttard L, Beresford B, Clarke S, Beecham J, Todd S, Bromley J. Riding the Rapids: Living with autism or disability—An evaluation of a parenting support intervention for parents of disabled children. Research in Developmental Disabilities. 2014 Oct 1;35(10):2371-83.

Self Management Empowerment Program.

1. Zare N, Ravanipour M, Bahreini M, Motamed N, Hatami G, Nemati H. Effect of a self-management empowerment program on anger and social isolation of mothers of children with cerebral palsy: A randomized controlled clinical trial. Evidence Based Care. 2017 Oct 1;7(3):35-44.

Single Session Therapy Approach.

1. Mulligan J, Olivieri H, Young K, Lin J, Anthony SJ. Single session therapy in pediatric healthcare: the value of adopting a strengths-based approach for families living with neurological disorders. Child and Adolescent Psychiatry and Mental Health. 2022 Jul 22;16(1):59.

Skippu Mama Program.

1. Niinomi K, Asano M, Kadoma A, Yoshida K, Ohashi Y, Furuzawa A, Yamamoto M, Yamakita N, Mori A. Developing the “Skippu‐Mama” program for mothers of children with autism spectrum disorder. Nursing & Health Sciences. 2016 Sep;18(3):283-91.

SMART and PACT Parent Skills Training Program. Family Based Therapy.

1. Family Based Therapy. SMART and PACT Parent Skills Training Program. [Internet]. UK;2025. [cited: February 2025]. Available from: <https://www.familybasedtherapy.co.uk/the-smart-parent-carer-skills-training-programme/>

SmartAutism App.

1. Bonnot O, Adrien V, Venelle V, Bonneau D, Gollier-Briant F, Mouchabac S. Mobile app for parental empowerment for caregivers of children with autism spectrum disorders: prospective open trial. JMIR Mental Health. 2021 Sep 15;8(9):e27803.

Social Skills training group (SSTG).

1. Weiss JA, Viecili MA, Sloman L, Lunsky Y. Direct and indirect psychosocial outcomes for children with autism spectrum disorder and their parents following a parent-involved social skills group intervention. Journal of the Canadian Academy of Child and Adolescent Psychiatry. 2013 Nov;22(4):303.

Social Support to Empower Parents (STEP).

1. Sullivan-Bolyai S, Bova C, Leung K, Trudeau A, Lee M, Gruppuso P. Social support to empower parents (STEP). The Diabetes Educator. 2010 Mar;36(1):88-97.

Stepping Stones - Triple P Program.

1. Sanders MR, Mazzucchelli TG, Studman LJ. Stepping Stones Triple P: The theoretical basis and development of an evidence‐based positive parenting program for families with a child who has a disability. Journal of Intellectual and Developmental Disability. 2004 Sep 1;29(3):265-83.
2. Whittingham K, Sofronoff K, Sheffield J, Sanders MR. Stepping Stones Triple P: An RCT of a parenting program with parents of a child diagnosed with an autism spectrum disorder. Journal of abnormal child psychology. 2009 May;37:469-80.
3. Theobald M, Aschersleben G, Karbach J, Hasmann R, Karpinski N, Petermann F. Children with disabilities and their families: Evaluation of Stepping Stones Triple P parental training. ZEITSCHRIFT FUR PSYCHIATRIE PSYCHOLOGIE UND PSYCHOTHERAPIE. 2015 Oct 1;63(4):247-53.
4. Schrott B, Kasperzack D, Weber L, Becker K, Burghardt R, Kamp-Becker I. Effectiveness of the stepping stones triple P group parenting program as an additional intervention in the treatment of autism spectrum disorders: effects on parenting variables. Journal of Autism and Developmental Disorders. 2019 Mar 15;49:913-23.
5. Sanders MR. Development, evaluation, and multinational dissemination of the Triple P-Positive Parenting Program. Annual review of clinical psychology. 2012 Apr 27;8(1):345-79.
6. Ruane A, Carr A, Moffat V. A qualitative study of parents’ and facilitators’ experiences of Group Stepping Stones Triple P for parents of children with disabilities. Clinical child psychology and psychiatry. 2019 Oct;24(4):694-711.
7. Hodgetts S, Savage A, McConnell D. Experience and outcomes of stepping stones triple P for families of children with autism. Research in developmental disabilities. 2013 Sep 1;34(9):2572-85.
8. Hinton S, Sheffield J, Sanders MR, Sofronoff K. A randomized controlled trial of a telehealth parenting intervention: A mixed-disability trial. Research in Developmental Disabilities. 2017 Jun 1;65:74-85.
9. Delach TB. Parent Perceptions of the Acceptability, Effectiveness, and Experience of Engaging in the Group Stepping Stones Triple P Intervention for Parents of Children with Disabilities. University of South Florida; 2020.
10. Brian J, Tint A, Branson JC, Pilkington M. Effectiveness of Group Stepping Stones Positive Parenting Program for children with autism spectrum disorder and disruptive behaviour: Program evaluation from a large community implementation. Journal on Developmental Disabilities. 2021 May 1;26(2):1-9.
11. Andersson E, McIlduff C, Turner K, Thomas S, Davies J, Elliott EJ, Einfeld S. Jandu Yani U ‘For All Families’ Triple P—positive parenting program in remote Australian Aboriginal communities: a study protocol for a community intervention trial. BMJ open. 2019 Oct 1;9(10):e032559.

Strong parents Strong children Program.

1. Jerram H, Raeburn J, Stewart A. The Strong Parents-Strong Children Programme: parental support in serious and chronic child illness. The New Zealand Medical Journal (Online). 2005 Oct 28;118(1224).

Surpassing Obstacles Autism Retreat (SOAR).

1. Williams PR. The Effects of Autism Training on Empowerment Outcomes for Parents and Caregivers of Autistic Youth. Drexel University; 2022.

Text2Dads.

1. May CD, St George JM, Lane S. Fathers raising children on the autism spectrum: lower stress and higher self-efficacy following SMS (Text2dads) intervention. Journal of autism and developmental disorders. 2022 Jan;52(1):306-15.

The A-PLUS Network.

1. Ireys HT, Sills EM, Kolodner KB, Walsh BB. A social support intervention for parents of children with juvenile rheumatoid arthritis: Results of a randomized trial. Journal of Pediatric Psychology. 1996 Oct 1;21(5):633-41.

The Empower Us Project. Koala

1. Koala. The Empower Us Project. [Internet]. UK; 2025. [cited: February 2025]. Available from: <https://koalanw.co.uk/service/empower-us-project>

The Empowerment Education Program.

1. Askari S, Hassanpour T, Shirvani Z, Eslamiyan S, Gholamnezhad M. Health Education and quality of life of mothers with mentally retarded daughters. NeuroQuantology. 2022 Dec; 20(16): 4900-4904.

The Expert Parent Program. Council for Disabled Children

1. The Council for Disabled Children. The Expert Parent Program. UK; 2025. [cited February, 2025]. Available from: <https://councilfordisabledchildren.org.uk/what-we-do-0/practice/health-wellbeing/health-wellbeing-practice/expert-parent-programme>

The Parent Child Relationally informed - Early Intervention.

1. Callanan J, Ronan K, Signal T. Activating parents in early intervention: Preliminary findings from an empirical case study. International Journal of Disability, Development and Education. 2020 Jan 2;67(1):1-7
2. Callanan J, Signal T, McAdie T. What is my child telling me? Reducing stress, increasing competence and improving psychological well-being in parents of children with a developmental disability. Research in developmental disabilities. 2021 Jul 1;114:103984.

The Veteran Parent Program.

1. Baron Nelson M, Riley K, Arellano K. Adding a parent to the brain tumor team: Evaluating a peer support intervention for parents of children with brain tumors. Journal of Pediatric Oncology Nursing. 2018 May;35(3):218-28.

Time out for Parents: Children with Additional Needs. Care for the Family.

1. Care for the Family. Time out for Parents: Children with Additional Needs. [Internet]. UK; 2025. [cited: February 2025]. Available from: <https://www.careforthefamily.org.uk/courses/courses-for-parents/parenting-courses-time-out/time-out-for-parents-children-with-special-needs/>

Tomorrow 's Challenge.

1. Hixson DD, Stoff E, White PH. Parents of children with chronic health impairments: A new approach to advocacy training. Children's Health Care. 1992 Mar 1;21(2):111-5.

Ubuntu/Baby Ubuntu (previously Getting to know Cerebral palsy)

1. Zuurmond M, O’Banion D, Gladstone M, Carsamar S, Kerac M, Baltussen M, Tann CJ, Gyamah Nyante G, Polack S. Evaluating the impact of a community-based parent training programme for children with cerebral palsy in Ghana. PloS one. 2018 Sep 4;13(9):e0202096.
2. Zuurmond M, Seeley J, Shakespeare T, Nyante GG, Bernays S. Illuminating the empowerment journey of caregivers of children with disabilities: Understanding lessons learnt from Ghana. African Journal of Disability. 2020 Nov 27;9:705.
3. Nanyunja C, Sadoo S, Kohli-Lynch M, Nalugya R, Nyonyintono J, Muhumuza A, Katumba KR, Trautner E, Magnusson B, Kabugo D, Cowan FM. Early care and support for young children with developmental disabilities and their caregivers in Uganda: The Baby Ubuntu feasibility trial. Frontiers in Pediatrics. 2022 Sep 13;10:981976.

Volunteer Advocacy Program - Transition (VAP-T).

1. Taylor JL, Hodapp RM, Burke MM, Waitz-Kudla SN, Rabideau C. Training parents of youth with autism spectrum disorder to advocate for adult disability services: Results from a pilot randomized controlled trial. Journal of autism and developmental disorders. 2017 Mar;47:846-57.

Web based epilepsy education program (WEEP).

1. Güven ŞT, Dalgiç Aİ, Duman Ö. Evaluation of the efficiency of the web-based epilepsy education program (WEEP) for youth with epilepsy and parents: A randomized controlled trial. Epilepsy & Behavior. 2020 Oct 1;111:107142.

Wise Up Workshops. Somerset Parent Carer Forum.

1. Somerset Parent Carer Forum. Wise Up Workshops. [Internet]. UK; 2025. [cited February 2025]. Available from: <https://somersetparentcarerforum.org.uk/home/wise-up-workshops/>

World Health Organisation Parent Skills Training (WHO-PST).

1. Salomone E, Pacione L, Shire S, Brown FL, Reichow B, Servili C. Development of the WHO caregiver skills training program for developmental disorders or delays. Frontiers in psychiatry. 2019 Nov 11;10:769.
2. Hamdani SU, Huma ZE, Suleman N, Akhtar P, Nazir H, Masood A, Tariq M, Koukab A, Salomone E, Pacione L, Brown F. Effectiveness of a technology-assisted, family volunteers delivered, brief, multicomponent parents’ skills training intervention for children with developmental disorders in rural Pakistan: a cluster randomized controlled trial. International journal of mental health systems. 2021 May 31;15(1):53.
3. Hamdani SU, Akhtar P, Nazir H, Minhas FA, Sikander S, Wang D, Servilli C, Rahman A. WHO Parents Skills Training (PST) programme for children with developmental disorders and delays delivered by Family Volunteers in rural Pakistan: study protocol for effectiveness implementation hybrid cluster randomized controlled trial. Global mental health. 2017 Jan;4:e11.

WWW-Roadmap (What, Where, Who).

1. Alsem MW, Van Meeteren KM, Verhoef M, Schmitz MJ, Jongmans MJ, Meily-Visser JM, Ketelaar M. Co-creation of a digital tool for the empowerment of parents of children with physical disabilities. Research involvement and engagement. 2017 Dec;3:1-2.
2. Alsem MW, Verhoef M, Braakman J, van Meeteren KM, Siebes RC, Jongmans MJ, Visser‐Meily JM, Ketelaar M, WWW, roadmap study group. Parental empowerment in paediatric rehabilitation: exploring the role of a digital tool to help parents prepare for consultation with a physician. Child: care, health and development. 2019 Sep;45(5):623-36.
3. Alsem MW, Van Meeteren KM , Siebes RC, Meily-Visser JM, Jongmans MJ , Verhoef M, Ketelaar M. Empowerment of parents of children with disabilities: results of the use of an online information tool as preparation for consultation with a rehabilitation physician. Developmental Medicine and Child Neurology. 2018 Vol. 60(Supplement 2) Pages 8.
