# Supplementary material for: Interventions supporting the empowerment of parent carers of children with neurodisability and other long‐term health conditions: A scoping review
Source: Dev Med Child Neurol. 2025 Oct 26;68(4):489–500. doi: 10.1111/dmcn.70039 (PMC12982629; doi:10.1111/dmcn.70039)
Supplement: Supplementary file 6 — Appendix S5: Using the database. [file DMCN-68-489-s004.docx]

## Appendix S5– Using the Database

3


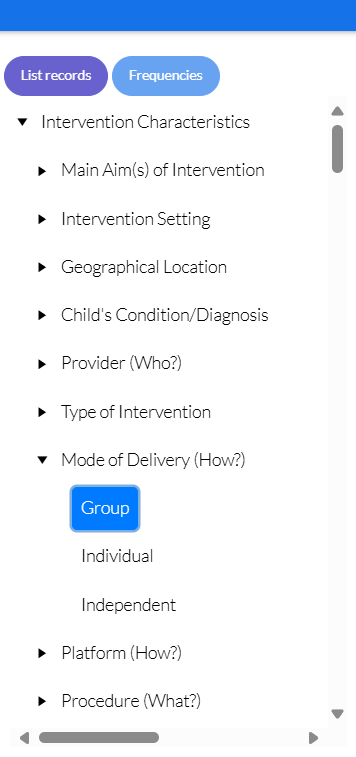


In the database, data is presented in **categories** and **codes**.

**Categories** are presented alongside a small black arrow. **Codes** appear beneath the category when it has been opened.

It is possible to explore the database in a number of ways:

**1) Listing all records linked to a particular code.**

To do this, in the box on the left of the screen, **open** a category by clicking on the **small black arrow**.^1^ Next, **highlight** a single code within that category by **clicking on the code** and **turning it blue**.^2^  Finally click the ‘List Records’ button.^3^  You will then be presented with a list of all interventions that have been assigned that code.

2

1

e.g. If you select ‘Mode of delivery’ > ‘Group’, then click ‘List Records’, you will be presented with all the interventions that are delivered to a group.

**2) Checking frequency data.**

You can also look at how data is spread between the codes in any given category.  To do this, **highlight** a category by **clicking on the category** and **turning it blue**.^1^ Then click the ‘Frequencies’ button.^2^  This data will then be presented in the ‘Frequencies’ box below.  You can chose for the data to be presented as a table or in a bar or pie chart.

**
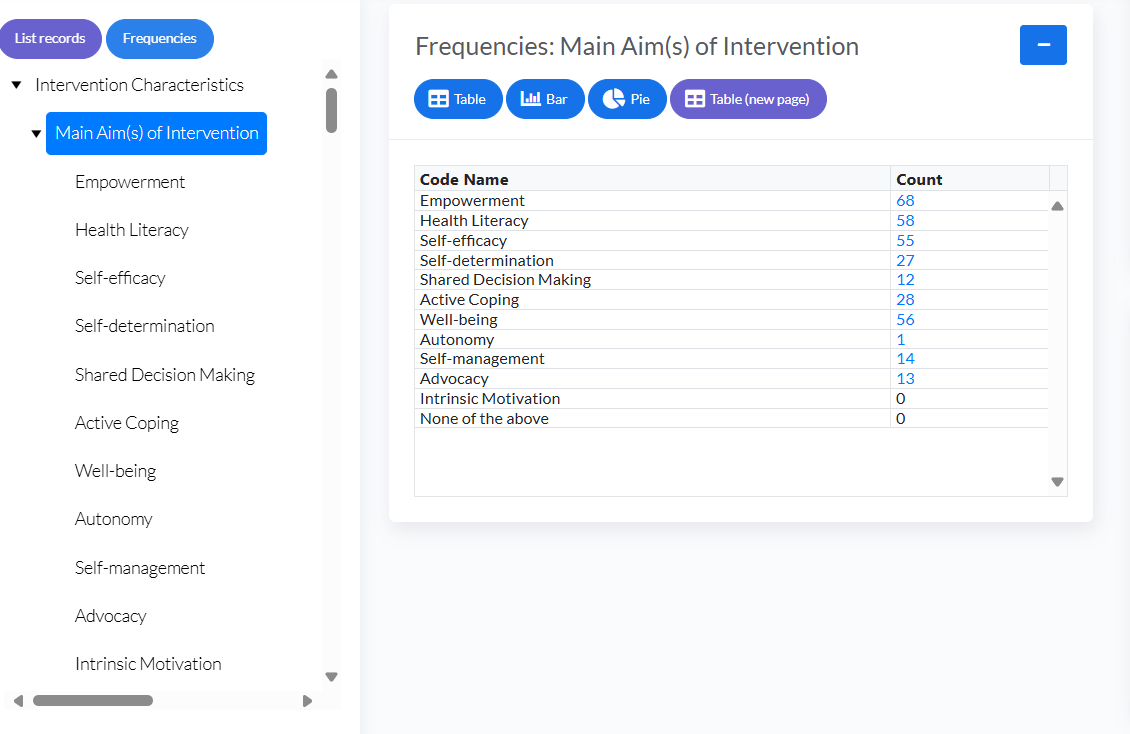
**e.g. If you select ‘Main Aim(s) of Intervention’ category, then click ‘Frequencies’, you will be presented with a table of all the codes in that category and their frequencies. You can click on the blue number to list all associated records.^3^

2

1

3

**3) Cross checking data.**

^
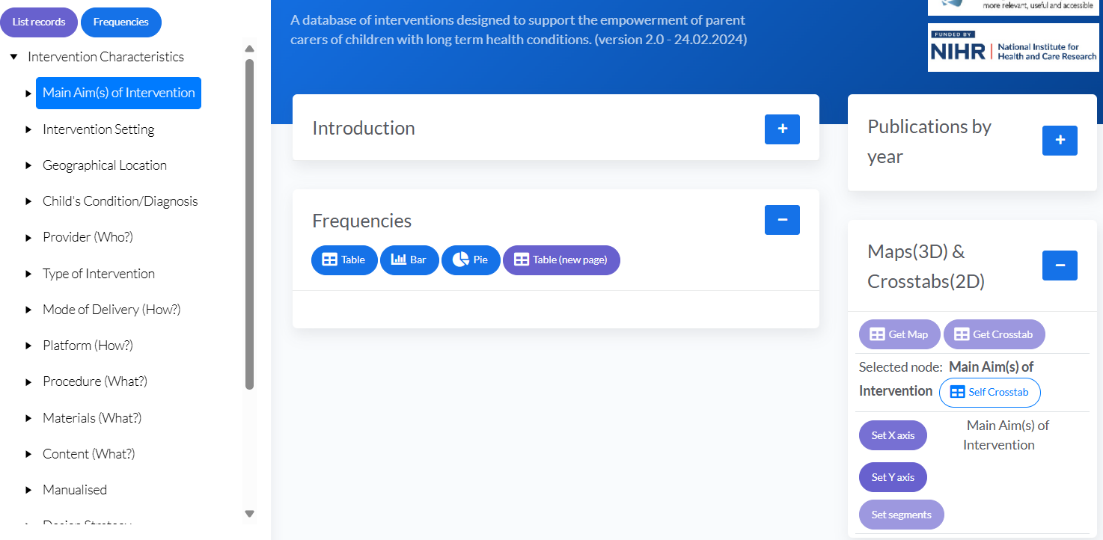
^In addition, you can map one category against another using the 'Crosstabs/Maps' box in the bottom right of the screen.  This allows you to see frequency data across two categories.  To do this, **highlight the first category**^1^ as before, then click the ‘set as x axis’ button.^2^  Next, **highlight the second category**^3^ and click the ‘set as y axis’ button.^4^  Finally, click the ‘get crosstabs’ button.^5^

1

2

**
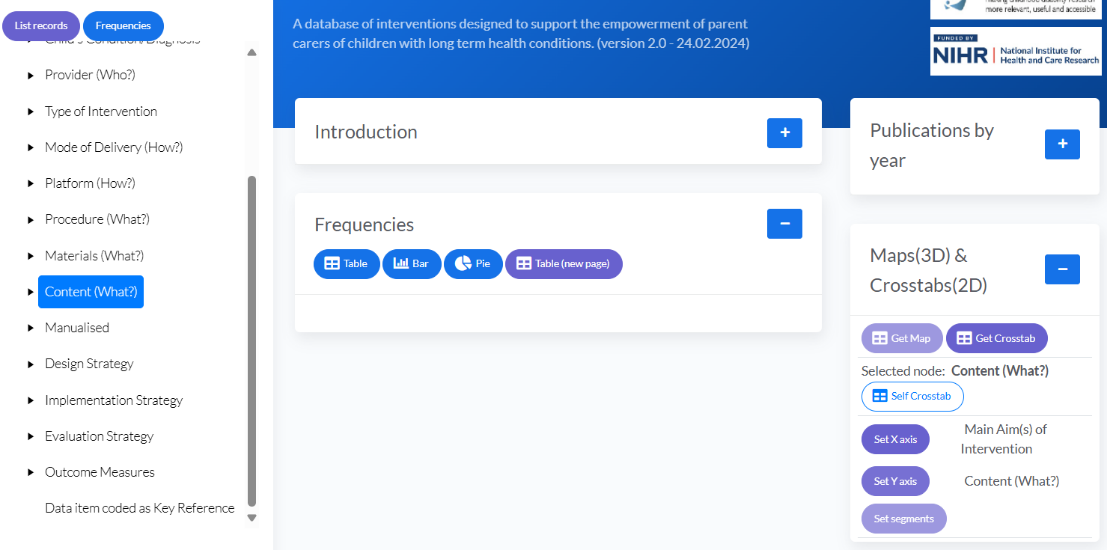
**

3

5

4

**
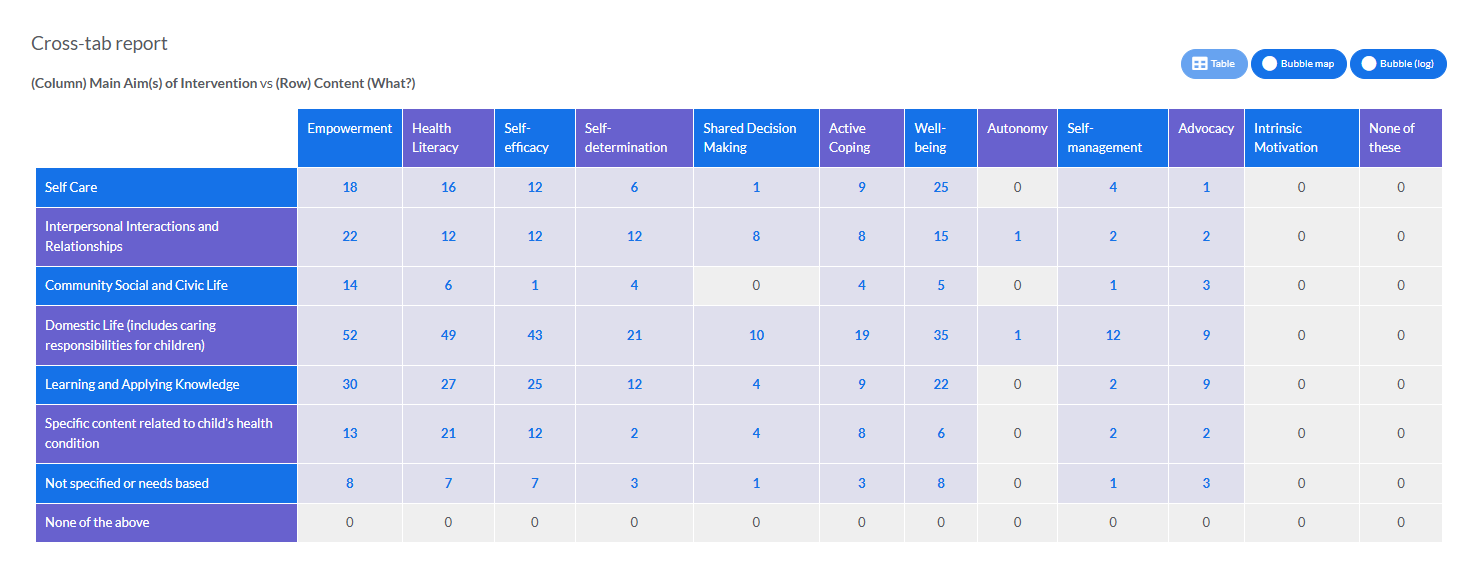
**This is an example of a cross-tab report for ‘Main Aim(s) of Intervention’ and ‘Content’. Records with both codes (e.g. ‘Learning and Applying Knowledge’ and ‘Self Efficacy’ can be accessed by clicking on the blue number.^6^

6

**Accessing the Information Source:**

You can access all the available data for each intervention by clicking on the information source.  This will show imported data and a coding report. There are also links to any additional sources of information about the intervention identified in the original searches recorded in the comments box.

**Worked Example:**

Searching for all interventions evaluated using and ‘Randomised Controlled Trial’ with the ‘Family Empowerment Scale’.

1. Using the ‘Cross-Tab’ Feature.
2. Highlight the ‘Evaluation Strategy’ category.
3. Set to the x-axis.
4. Highlight the ‘Outcome Measure’ category.
5. Set to the y-axis.
6. Click ‘Get Cross-Tab’
7. On the Cross-tab report, scroll down to find the RCT x FES box and click on the blue number.

A list of all the interventions that used and RCT ‘AND’ the Family Empowerment Scale will be created. Each intervention can then be opened to access imported data and the coding report.
