# Supplementary material for: Interventions supporting the empowerment of parent carers of children with neurodisability and other long‐term health conditions: A scoping review
Source: Dev Med Child Neurol. 2025 Oct 26;68(4):489–500. doi: 10.1111/dmcn.70039 (PMC12982629; doi:10.1111/dmcn.70039)
Supplement: Supplementary file 7 — Figure S1: PRISMA flow chart. [file DMCN-68-489-s008.pdf]

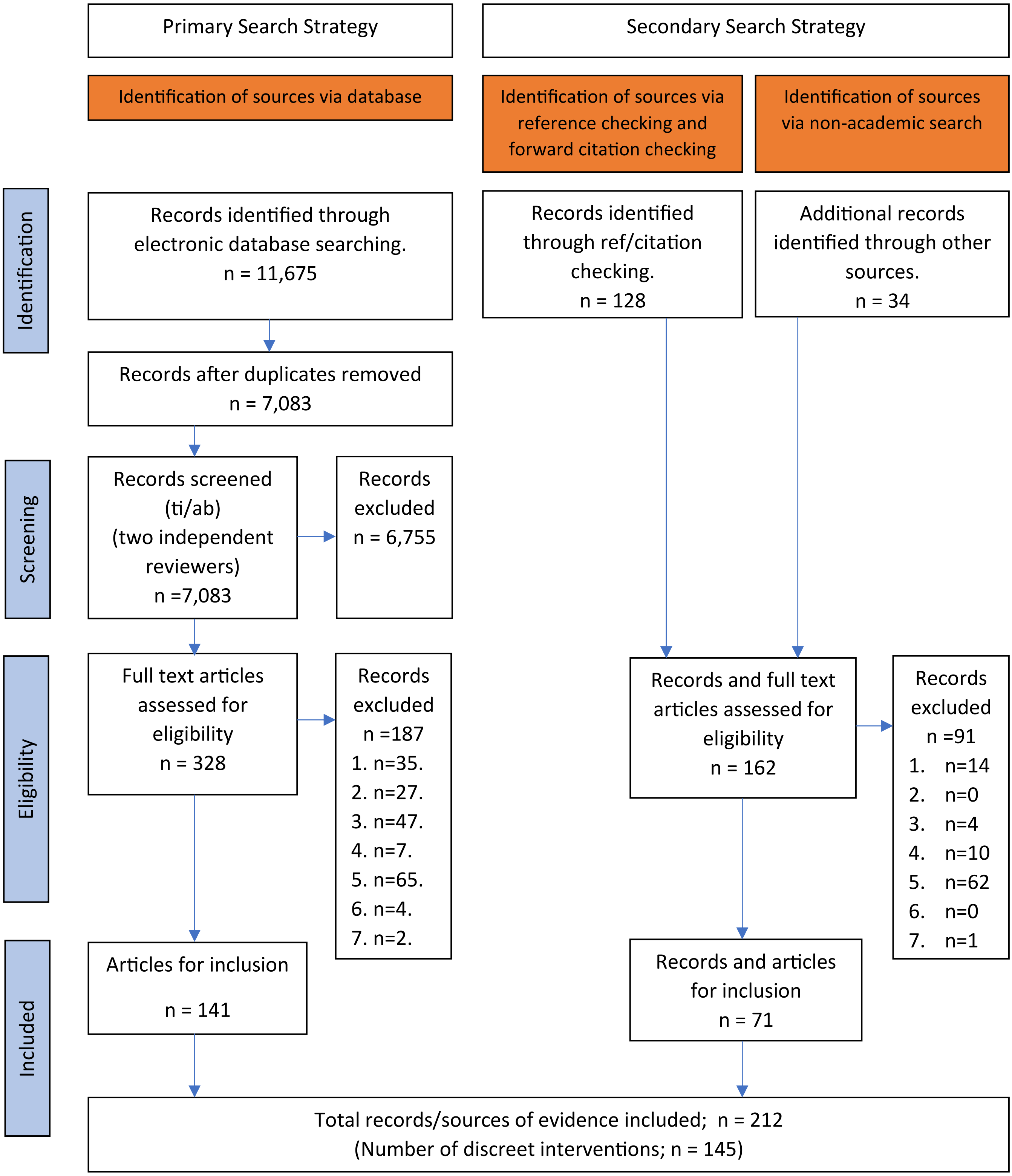

Reasons for exclusion:

1. Reports on an identified intervention (no additional data to extract).
2. Limited information to extract - secondary search did not retrieve more information.
3. No specific intervention is described
4. Children do not have a long term health condition.
5. Focus of intervention is not listed in eligibility criteria.
6. Unable to retrieve information.
7. Intervention delivered in an Acute Setting
